# Alternation of gene expression in brain-derived exosomes after cerebral ischemic preconditioning in mice

**DOI:** 10.1016/j.heliyon.2024.e35936

**Published:** 2024-08-08

**Authors:** He Li, Xiaoxi Zhang, Hongye Xu, Hanchen Liu, Yongxin Zhang, Lei Zhang, Yu Zhou, Yongwei Zhang, Jianmin Liu, Mei Jing, Ping Zhang, Pengfei Yang

**Affiliations:** a Emergency Department, Naval Medical Center of PLA, Naval Medical University, Shanghai, China; b Neurovascular Center, Changhai Hospital, Naval Medical University, Shanghai, China; c Department of Neurology, Naval Medical Center of PLA, Naval Medical University, Shanghai, China

**Keywords:** Cerebral ischemic preconditioning, Cerebral ischemia-reperfusion injury, Exosomes, Neuroprotection, Whole transcriptome sequencing

## Abstract

**Aims:**

Cerebral ischemic preconditioning is a neuroprotective therapy against cerebral ischemia and ischemia-reperfusion injury. This study aims to demonstrate the alternation of gene expression in exosomes from brain tissue of mice after ischemic preconditioning and their potential functions.

**Methods:**

Ten mice were divided into the sham and the cerebral ischemic preconditioning groups. Their brain tissues were harvested, from which the exosomes were extracted. The characteristics and protective effects of exosomes were evaluated. Whole transcriptome sequencing was used to demonstrate the gene expression discrepancy between the exosomes from the two groups of mice brains. Volcano graphs and heatmaps were used to picture the difference in expression quantity of mRNA, lncRNA, and circRNA. Gene ontology (GO) analysis and Kyoto Encyclopedia of Genes and Genomes (KEGG) pathway analysis were performed to demonstrate the functions of differentially expressed RNAs.

**Results:**

Exosomes were successfully extracted, and those from the cerebral ischemic preconditioning group had better protective effects on cells that received oxygen-glucose deprivation and restoration injury. A total of 306 mRNAs and 374 lncRNAs were significantly upregulated, and 320 mRNAs and 405 lncRNAs were significantly downregulated in the preconditioning group. No circRNAs were differentially expressed between the two groups. GO and KEGG pathway analysis indicated that the functions of differentially expressed RNAs were related to both neural protective and injurious effects.

**Conclusion:**

The brain-derived exosomes may participate in the neuroprotective effect of cerebral ischemic preconditioning. Thorough research is necessary to investigate exosome functions derived from the ischemic preconditioned brain.

## 1 Introduction

Ischemic stroke is a leading cause of death and imposes a significant burden on global health. The prevalence of ischemic stroke is 2.5 %, and 150,000 deaths are caused by ischemic stroke annually [1]. Although the development of techniques, including thrombolysis and endovascular thrombectomy, has increased the vascular recanalization rate of ischemic stroke, the clinical outcomes of a large proportion of ischemic strokes remain unsatisfactory [2]. Cerebral ischemia-reperfusion (I/R) injury, one of the major pathological changes after vascular recanalization in ischemic stroke, hampers the achievement of good outcomes [3]. Many investigators have endeavored to identify novel neuroprotective methods to mitigate the pathological cascade after reperfusion, but they still fail to translate their findings into clinical practice [4].

These years, the neuroprotective value of ischemic preconditioning (IPC) has been revealed by several studies [5,6]. Remote ischemic preconditioning (RIPC) can generate repeated intermittent limb ischemia, thereby reducing the incidence of stroke in patients with cerebrovascular stenosis [7]. On the other hand, patients with chronic cerebrovascular stenosis or transient ischemic attack (TIA) history usually have moderate symptoms and smaller infarct volume than those without a history of ischemic stroke, which provides solid evidence for the existence of protective in situ cerebral ischemia preconditioning (CIPC) [8]. However, the major limitation of IPC is that effective IPC cannot be produced at the time of ischemic stroke onset. The potential side effect is another barrier to translating IPC from the bench to the bedside. Thus, it is necessary to thoroughly investigate the mechanism of CIPC and identify its protective aspect to avoid injury.

Recently, the role of exosomes in IPC has been illustrated [[9], [10], [11]]. The exosome is an extracellular vesicle with a 50–150 nm diameter containing bioactive molecules, including proteins and nucleotides [12,13]. Xun-Ming Ji and colleagues leadingly reported the neuroprotective effect of exosomes containing hypoxia-inducible transcription factor (HIF)-1α induced by RIPC [14]. Our team illustrated that exosomes containing miR-451a are involved in the protective effects of CIPC [15]. These studies highlight the potential therapeutic value of IPC-induced exosomes (IPC-Exosomes, IPCE) in dealing with I/R injury. However, current research has identified a few protective molecules in IPCE. The protective effect of IPCE may be generated by a bulk of neuroprotective molecules that work synergistically or individually. Thus, the mechanisms underlying the IPCE require further investigation.

To comprehensively study the effective components of IPCE, we performed whole-transcriptome sequencing (WTS) using exosomes derived from the brain tissue of mice that received in situ CIPC. We identified several differentially expressed mRNAs and lncRNAs potentially related to the biological functions of IPCE, according to the WTS atlas. Gene Ontology (GO) and Kyoto Encyclopedia of Genes and Genomes (KEGG) pathway analyses were also performed to demonstrate the potential functions of the differentially expressed nucleotides.

## 2 Methods

### 2.1 Experiment workflow

**Fig. 1:**
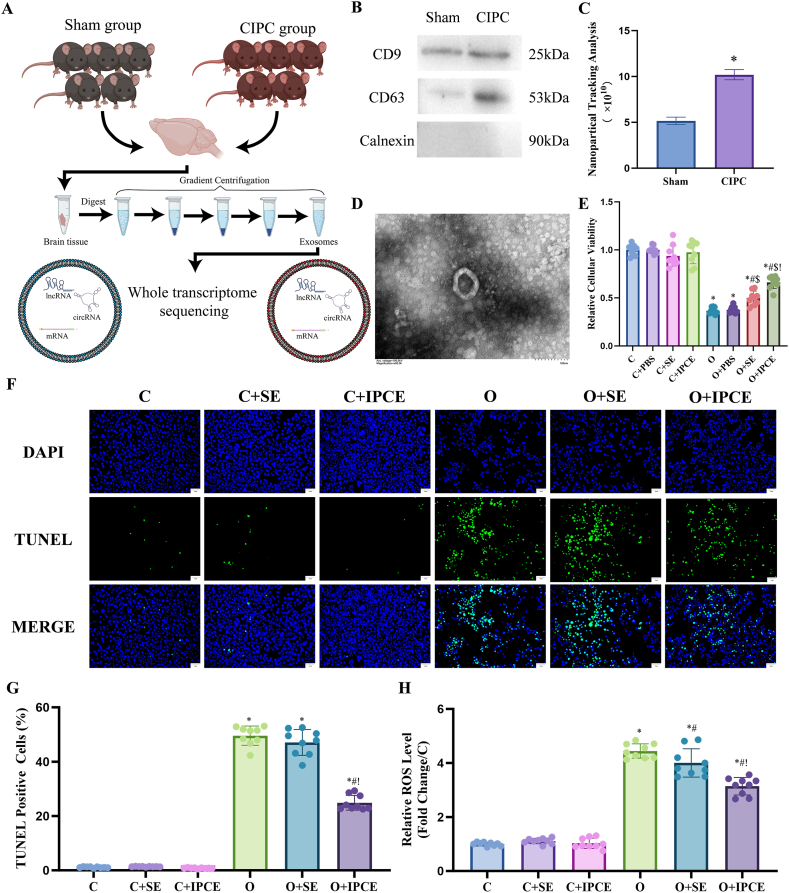
(A) Workflow of exosome extraction and the WTS. (B) Western blot analysis of exosomal markers. (C) NTA of the exosomes. (D) TEM observation of the exosomes. (E) Relative viability of each cell line. (F and G) TUNEL staining in each cell group. DAPI-positive and TUNEL-positive cells were detected using 470 nm and 364 nm excitation lasers, respectively. (H) Relative ROS levels in each cell group. (C: Control; C + PBS: Control + PBS; C + SE: Control + exosomes from the Sham group mice; C + IPCE: Control + exosomes from the CIPC group mice; O: OGD/R; O + PBS: OGD/R + PBS; O + SE: OGD/R + exosomes from the Sham group mice; O + IPCE: OGD/R+ exosomes from the CIPC group mice; *, #, $, and! represented p < 0.05 compared to the C, O, O + PBS, and O + SE groups.)

Ten C57BL/6 mice (male, 8 weeks old, 25 ± 2 g; Charles River Laboratories) were divided into two groups: the Sham and the CIPC. The brains were harvested 24 h after the operation and stored in a refrigerator at −80 °C. The brain tissue was homogenized, digested, and subjected to gradient centrifugation to extract exosomes. WTS analyzed the exosomes to identify differentially expressed nucleotides (Fig. 1A). The animal study was approved by the ethics committee of Changhai Hospital (CH20220310). All animal experiments were performed in accordance with the National Institute of Health's Guide for the Care and Use of Laboratory Animals.

### 2.2 CIPC models

Mice in the CIPC group underwent two cycles of bilateral common carotid artery occlusion (BCCAO) for 5 min, as previously described [15,16]. Briefly, a medial neck incision was made in each mouse and the bilateral common carotid arteries were exposed. BCCAs were clipped using artery clamps for 5 min, and the same operation was repeated after a 15-min gap. Mice in the sham group underwent a medial neck incision and bilateral common carotid artery explosion, without BCCAO.

### 2.3 Extraction of exosomes from brain tissue

Brain cortices were harvested from the two groups of mice. Briefly, mice were sacrificed by CO2 asphyxiation following cerebral perfusion with phosphate-buffered saline (PBS). After exposing the skull, a midline incision was made to expose the brain. The meninx was removed from the brain tissue and the brain tissue was separated from the skull base using a nerve dissector.

Subsequently, brain-derived exosomes were extracted using a previously described method [17]. Briefly, the brain tissues were homogenized in PBS using a homogenizer and centrifugated at 1200r/min for 5 min at 4 °C. The supernatant was removed and collagenase IV working solution was added to the residue. The residue was shaken at 90r/min for 60 min at 37 °C. The supernatant was collected and centrifuged at 2000×*g* for 10 min and 10,000×*g* for 30 min at 4 °C. The supernatant was then ultracentrifuged at 110,000×*g* for 75 min at 4 °C and discarded. After resuspension and filtration, the residue was subjected to ultracentrifugation at 110,000 g/min for 75 min at 4 °C. The exosomes in the residue were resuspended in PBS and stored at −80 °C.

### 2.4 Characterization of exosomes

Western blotting was used to identify the exosome markers. CD9 (CST, 98327) and CD63 (Abcam, ab217345) were used to mark exosomes and calnexin (Abcam, ab133615) antibodies were used as negative markers of exosomes. Briefly, exosomal proteins were extracted using RIPA Lysis Buffer (Beyotime, P0013B), according to the manufacturer's instructions. Ten micrograms of protein per sample were separated using 10 % ExpressPlus™ Gels (GenScript, USA) and then transferred to polyvinylidene fluoride (PVDF) membranes. After blocking with QuickBlock Blocking Buffer (Beyotime, P0252), the membranes were incubated overnight at 4 °C with antibodies. The membranes were washed and incubated with secondary antibodies for 2 h, and positive bands were detected using BeyoECL Plus (Beyotime, P0018S) developing agent. Nanoparticle tracking analysis (NTA) was performed to evaluate exosome size distribution and concentration. NTA was performed using a ZetaVIEW S/N 17–310 machine and measured using ZetaView 8.04.02. Transmission electron microscopy (TEM) was performed using a Hitachi (HT7700) TEM to examine the morphology of individual exosomes.

### 2.5 Verify the function of the brain-derived exosomes

To investigate the function of exosomes, we administered exosomes to Neuro2a (N2a) cells. N2a cells were cultured in MEM (ZQ-300, Zhong Qiao Xin Zhou) supplemented with 10 % fetal bovine serum (FBS, Zhong Qiao Xin Zhou) and 1 % penicillin-streptomycin (0513, ScienCell) under 5 % CO2. The cells were embedded in 96-well plates and divided into eight groups: C (control), C + PBS (Control + PBS), C + SE (control + exosomes from the sham group mice), C + IPCE (control + exosomes from the CIPC group mice), O (oxygen-glucose deprivation and restoration, OGD/R), O + PBS, O + SE, and O + IPCE. Cell Counting Kit-8 (CCK-8) was used to evaluate the viability of each group. The numbers of cells seeded in 96-pore plates and 6-pore plates for different purposes were 5 × 10^4^ and 1 × 10^6^, respectively. The final concentration of exosomes in the treated groups was approximately 1 × 10^11^ particles/ml.

The OGD/R model was established as previously described [18]. Briefly, the medium was replaced with glucose-free Hanks’ balanced salt solution (Zhong Qiao Xin Zhou Co., Ltd., China) and the cells were incubated under 95 % N_2_ and 5 % CO_2_ for 4 h. After OGD, the cells were transferred to normal serum-free conditions and cultured for 20 h before further measurements.

Cell Counting Kit-8 (CCK-8) (HY–K0301, MedChemExpress), TUNEL staining of cells (C1086, Beyotime), and reactive oxidative species (ROS) testing assay (S0033, Beyotime) were used to evaluate the viability, apoptosis, and oxidative stress levels of the cells under different conditions. All tests were performed in accordance with the manufacturer's instructions.

### 2.6 Quantitative real-time PCR

mRNA and lncRNA levels in exosomes or cells were measured using quantitative real-time PCR (qRT-PCR). Total RNA was extracted using TRIzol reagent according to the manufacturer's instructions. The purity of the RNA samples was measured using the OD260/280 method, and the reverse transcribed method was used using the PrimeScriptTM RT reagent Kit (RR037A, TAKARA). qRT-PCR was performed on a LightCycler 96 (Roche Group) using SYBR® Premix Ex Taq™ II (Tli RNaseH Plus) (RR420A, TAKARA), and the fold-change in expression levels was calculated using the 2^-△△Ct^ method. Primer sequences were listed in Supplementary Fig. 1.

### 2.7 WTS analysis of exosomes

Exosomal RNA was extracted using an exoRNeasy Maxi kit (Qiagen), according to the manufacturer's protocol. The rRNA was dropped, and the remaining RNA was used to build the cDNA library. After quality control, the library was subjected to Illumina sequencing using the paired-end 150 strategy. Sequencing was performed in a sequencing-by-synthesis manner. Raw sequencing data were cleaned, and quantitative analysis of mRNA, lncRNAs, and circRNAs was performed using clean data. The target mRNAs of lncRNAs were predicted based on trans-acting and cis-acting mechanisms [19].

### 2.8 Data analysis

R studio was used to plot the volcano graph and heatmap of the comparison analysis of differentially expressed RNAs. GO and KEGG pathway analyses were also performed in R studio as previously described [20]. The results of GO analysis were divided into three parts: biological process (BP), molecular function (MF), and cellular component (CC). The top 20 gene functions of each category that enriched the most mRNAs were presented separately as bar plots. The top 20 pathways with the highest GeneRatio values were presented as dotted plots. P < 0.05 was considered statistically significant.

## 3 Results

### 3.1 The characterization of exosomes and their neuroprotective effects

Western blotting analysis indicated that the expression of exosomal biomarkers per unit weight of brain tissue, including CD9 and CD63, was upregulated in the CIPC group (Fig. 1B). Accordingly, NTA showed that the concentration of exosomes in the CIPC group was significantly higher than that in the sham group (Fig. 1C). TEM observations demonstrated the shape and size of the extracted particles, which proved that the morphological characteristics of the particles were in accordance with the features of exosomes (Fig. 1D).

According to the in vivo study, OGD/R injury impaired the viability of N2a cells (C vs. O: 1.00–0.05 vs. 0.37–0.03, p < 0.001), and SE, IPCE attenuated the impairment induced by OGD/R (O vs. O + IPCE: 0.37–0.03 vs. 0.50–0.07, p = 0.006; O vs. O + IPCE: 0.37–0.03 vs. 0.66–0.06, p < 0.001). IPCE had a better protective effect on impaired cells than SE (O + SE vs. O + IPCE: 0.50–0.07 vs. 0.66–0.07, p = 0.003) (Fig. 1E). TUNEL analysis indicated that IPCE, but not SE, reduced the proportion of TUNEL-positive cells after OGD/R injury (O vs. O + IPCE: 49.6 %–1.18 % vs. 24.9 %–0.91 %, p < 0.001) (Fig. 1F and G). While, the ROS testing assay showed that both SE and IPCE reduced the levels of ROS after OGD/R injury (O vs. O + SE:4.44–0.09 vs. 4.00–0.18, p = 0.027; O vs. O + IPCE: 4.44–0.09 vs. 3.14–0.11, p < 0.001), and the protective effect of IPCE was better than SE (Fig. 1H).

### 3.2 Differentially expressed RNAs between the sham group and the CIPC group

**Fig. 2:**
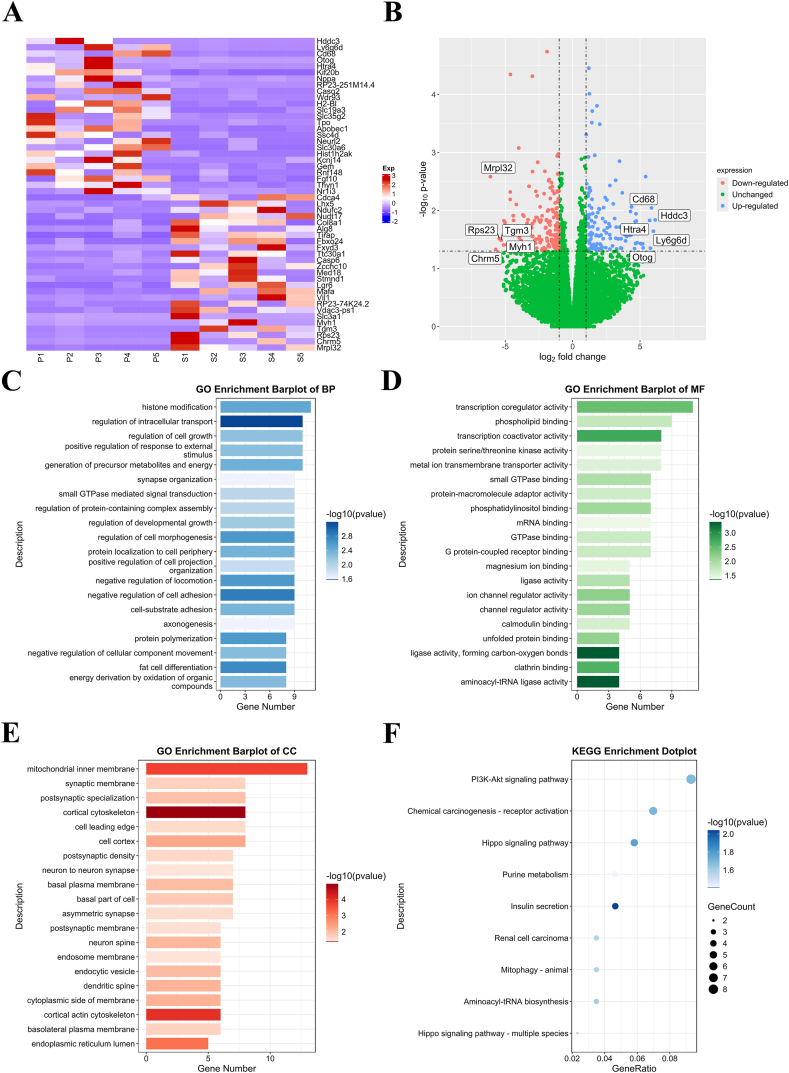
(A) Heatmap of differentially expressed mRNAs between exosomes from the Sham and the CIPC groups. (B) Volcano graph of differentially expressed mRNAs between exosomes from the Sham and the CIPC groups. (C–E) Bar plots of GO analysis of the upregulated mRNAs. (F) Dot plot of KEGG pathway analysis of the upregulated mRNAs.

According to the volcano plot, 306 mRNAs were significantly upregulated in the CIPC group, of which 203 mRNAs were upregulated more than twice (log_2_^Fold Change^ ≥ 1) compared to the Sham group. In contrast, 320 mRNAs were significantly downregulated in the CIPC group, including 241 mRNAs with log_2_^Fold Change^ ≤ −1 compared with the Sham group (downregulated more than two times) (Fig. 2A and B). The typical upregulated and downregulated mRNAs in the CIPC group were verified by qRT-PCR (Supplementary Fig. 1) and were listed in Table 1.

**Table 1:** The typical upregulated and downregulated mRNAs in IPCE.

mRNA names	Log2FC	p-value	UP-DOWN
Hddc3	6.1297	0.0145	UP
Ly6g6d	5.9892	0.0227	UP
Cd68	5.8552	0.009	UP
Otog	5.7686	0.0446	UP
Htra4	5.7146	0.0148	UP
Mrpl32	−6.0984	0.0026	DOWN
Chrm5	−5.706	0.0469	DOWN
Rps23	−5.4378	0.0307	DOWN
Tgm3	−5.3066	0.0325	DOWN
Myh1	−5.1203	0.0392	DOWN

In addition, 374 lncRNAs were significantly upregulated in the CIPC group, and all these lncRNAs were upregulated more than twice. In contrast, 405 lncRNAs were downregulated in the CIPC group, all of which were downregulated more than two times (Fig. 4A and B). The typical upregulated and downregulated mRNAs were verified by qRT-PCR (Supplementary Fig. 1) and were listed in Table 2.

**Table 2:** The typical upregulated and downregulated lncRNAs in IPCE

lncRNA names (IDs)	Log2FC	p-value	UP-DOWN
Tasp1-tv5	7.0763	0.0005	UP
Miat	6.9408	0.0107	UP
NONMMUT041264.2	6.6047	0.0158	UP
NONMMUT084500.1	5.9821	0.0035	UP
NONMMUT031944.2	5.9315	0.0282	UP
NONMMUT050671.2	−6.8563	0.0296	DOWN
ENSMUST00000172202	−6.8853	0.021	DOWN
NONMMUT016434.2	−6.9525	0.0001	DOWN
NONMMUT100091.1	−6.9553	0.0004	DOWN
NONMMUT007395.2	−7.2074	0.0044	DOWN

The expression levels of circRNAs were also compared. However, no circRNAs were significantly differentially expressed between the CIPC and Sham groups.

### 3.3 GO and KEGG pathway analyses of the upregulated mRNAs in the CIPC group

GO analysis of BP showed that the upregulated mRNAs were associated with histone modification, regulation of intracellular transport, generation of precursor metabolites and energy, positive regulation of response to external stimulus, and regulation of cell growth (Fig. 2C). GO analysis of the CC of the upregulated mRNAs indicated that these mRNAs were related to the mitochondrial inner membrane, cortical cytoskeleton, cell cortex, postsynaptic specialization, and synaptic membrane (Fig. 2D). For the MF analysis, the upregulated mRNAs were associated with transcription coregulator activity, phospholipid binding, transcription coactivator activity, metal ion transmembrane transporter activity, and protein serine/threonine kinase activity. (Fig. 2E).

KEGG pathway analysis indicated that the upregulated mRNAs in the CIPC group were enriched in pathways, including the PI3K-Akt signaling pathway, Chemical carcinogenesis-receptor activation, Hippo signaling pathway, Insulin secretion, and Purine metabolism (Fig. 2F).

### 3.4 GO and KEGG pathway analyses of the downregulated mRNAs in the CIPC group

**Fig. 3:**
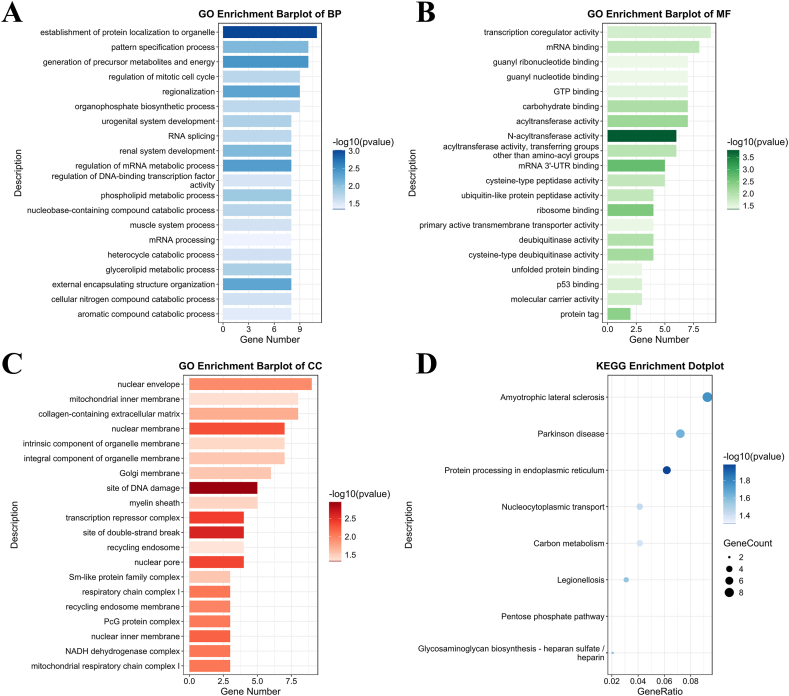
(A–C) Bar plots of GO analysis of downregulated mRNAs. (D) Dot plot of KEGG pathway analysis of the downregulated mRNAs.

GO analysis of BP indicated that the downregulated mRNAs participated in the establishment of protein localization to organelle, generation of precursor metabolites and energy, pattern specification process, regionalization, and regulation of mitotic cell cycle (Fig. 3A). GO analysis of the CC of the downregulated mRNAs indicated that these mRNAs were associated with the nuclear envelope, collagen-containing extracellular matrix, mitochondrial inner membrane, nuclear membrane, and integral component of organelle membrane (Fig. 3B). In the aspect of MF, the downregulated mRNAs were related to transcription coregulator activity, mRNA binding, acyltransferase activity, carbohydrate binding, and GTP binding (Fig. 3C).

**Fig. 4:**
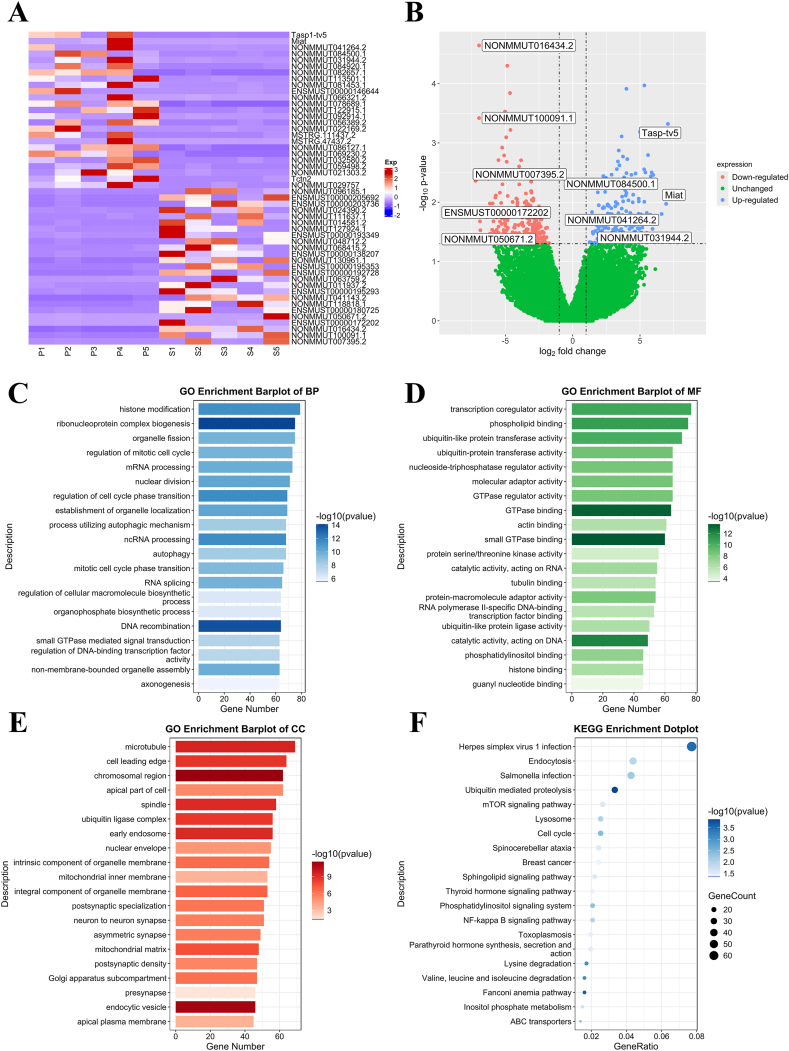
(A) Heatmap of differentially expressed lncRNAs between exosomes from the Sham and the CIPC groups. (B) Volcano graph of differentially expressed lncRNAs between exosomes from the Sham and the CIPC groups. (C–E) Bar plots of GO analysis of the target mRNAs of the upregulated lncRNAs. (F) Dot plot of KEGG pathway analysis of target mRNAs of upregulated lncRNAs.

KEGG pathway analysis demonstrated that the upregulated mRNAs were enriched in pathways, including Amyotrophic lateral sclerosis, Parkinson's disease, Protein processing in the endoplasmic reticulum, Nucleocytoplasmic transport, and Carbon metabolism (Fig. 3D).

### 3.5 The target prediction of differentially expressed lncRNAs

The target mRNAs of the differentially expressed lncRNAs were predicted by the cis- and trans-acting mechanisms. Totally 2195 mRNAs were predicted to be targets of the upregulated lncRNAs, and 2304 mRNAs were identified as targets of the downregulated lncRNAs in the CIPC group.

### 3.6 GO and KEGG pathway analyses of the target mRNAs of the upregulated lncRNAs in the CIPC group

GO analysis of BP showed that the target mRNAs were associated with histone modification, ribonucleoprotein complex biogenesis, organelle fission, mRNA processing, and regulation of mitotic cell cycle (Fig. 4C). CC analysis indicated that the target mRNAs were related to the microtubule, cell leading edge, chromosomal region, apical part of cell, and spindle (Fig. 4D). For MF, the target mRNAs were associated with transcription coregulator activity, phospholipid binding, ubiquitin-like protein transferase activity, ubiquitin-protein transferase activity, and molecular adaptor activity (Fig. 4E).

KEGG pathway analysis indicated that the target RNAs were enriched in pathways, including Herpes simplex virus 1 infection, Endocytosis, Salmonella infection, Ubiquitin mediated proteolysis, and mTOR signaling pathway (Fig. 4F).

### 3.7 GO and KEGG pathway analyses of the target mRNAs of the downregulated lncRNAs in the CIPC group

**Fig. 5:**
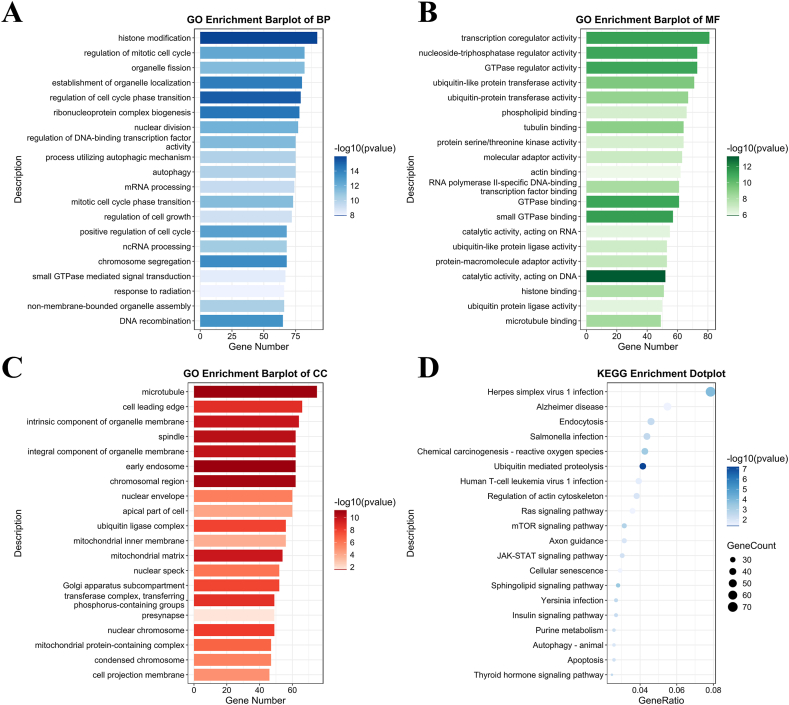
(A–C) Bar plots of GO analysis of the target mRNAs of the downregulated lncRNAs. (D) Dot plot of KEGG pathway analysis of target mRNAs of downregulated lncRNAs.

GO analysis of BP indicated that the target mRNAs were related to histone modification, regulation of mitotic cell cycle, organelle fission, establishment of organelle localization, and regulation of cell cycle phase transition (Fig. 5A). CC analysis showed that the target mRNAs were associated with microtubule, cell leading edge, intrinsic component of organelle membrane, early endosome, and chromosomal region (Fig. 5B). For MF, the target mRNAs were related to transcription coregulator activity, GTPase regulator activity, nucleoside-triphosphatase regulator activity, ubiquitin-like protein transferase activity, and ubiquitin-protein transferase activity (Fig. 5C).

KEGG pathway analysis illustrated that the target mRNAs were enriched in pathways, including Herpes simplex virus 1 infection, Alzheimer's disease, Endocytosis, Salmonella infection, and Chemical carcinogenesis-reactive oxygen species (Fig. 5D).

## 4 Discussion

This study is the first to present a comprehensive comparison of the whole transcriptome between exosomes from the brain tissue of mice undergoing sham operation and those receiving CIPC. The neuroprotective effects of the brain-derived IPCE were confirmed. We identified 306 upregulated mRNAs and 374 upregulated lncRNAs, and 320 mRNAs and 405 lncRNAs were significantly downregulated in IPCE compared to those in the Sham group. No circRNAs were differentially expressed between the CIPC and the Sham groups. GO and KEGG pathway analyses indicated that the differentially expressed mRNAs and the target mRNAs of the differentially expressed lncRNAs might participate in critical biological functions of exosomes during CIPC.

In recent years, progress in the clinical translation of RIPC has been boosted by the accomplishment of several clinical studies [7,21,22]. CIPC is also a promising neuroprotective method that has been proven in several experimental and clinical studies. However, the clinical translation of CIPC remains difficult because it is infeasible to actively induce CIPC in patients with acute ischemic stroke. Therefore, further experimental studies should thoroughly investigate the protective mechanisms of CIPC to identify new molecular targets for clinical translation. In our previous study, exosomes from the plasma of CIPC mice were shown to be neuroprotective [15]. In this study, brain-derived exosomes from CIPC mice were potentially neuroprotective against OGD/R. Therefore, investigating the components of these exosomes is necessary for further investigation of their functions.

According to our WTS analysis, several mRNAs that were upregulated in the CIPC group were neuroprotective. Kif20b (log2FC = 5.57) positively regulated cell proliferation and cytokinesis. Several studies illustrated the promotive effect of Kif20b on the proliferation and polarization of neurons, which might give rise to neuroprotective effects [23,24]. The upregulated Nppa (log2FC = 5.23) encoded a natriuretic peptide that controls extracellular fluid volume and electrolyte homeostasis. Nppa exerted protective effects on cardiomyocytes by stimulating autophagy by activating transcription factor EB [25]. Fgf10 (log2FC = 3.49) was also upregulated in the CIPC group. It was reported to activate the FGFR2-PI3K-Akt pathway in neurons and inhibit the TLR4/NF-κB pathway in microglia after spinal cord injury, exerting neuroprotective effects [26]. In contrast, several upregulated mRNAs also exerted injurious effects. Ets1 (log2FC = 1.56) encoded a DNA-binding transcription factor that participated in neuroinflammation and neuronal apoptosis, and it was upregulated in IPCE [27]. Piezo2 (log2FC = 1.52), which encoded a mechanosensitive ion channel component, was upregulated in IPCE. It was reported that Piezo2 exacerbated traumatic brain injury by activating RhoA/ROCK1 pathways [28]. Upregulated Trpv4 (log2FC = 1.28), which encoded a Ca^2+^ channel that mediates Ca^2+^ influx into the cell during ischemic stroke, exerted injurious effects [29]. Similarly, the downregulated mRNAs have different functions. A downregulated mRNA named Mrpl32 (log2FC = −6.10) encoded a ribosomal protein located in the mitochondrion, which was predicted to participate in OGD/R-induced apoptosis of neural cells [30]. Casp6 (log2FC = −4.18) encoded a cysteine protease that contributes to apoptosis and neurodegeneration [31]. Tirap (log2FC = −4.00) encoded a Toll-like receptor 2 binding protein that positively regulated the activity of the NF-κB transcription factor in cerebral I/R injuries [32]. In contrast, Lgr6 (log2FC = −4.61), an mRNA encoding a protein that attenuated neuroinflammation, was downregulated in IPCE [33]. Stmnd1 (log2FC = −4.53) encoded a protein with tubulin-binding activity, which was predicted to be active in the cytoplasm and neuron projections [34]. Lhx5 (log2FC = −3.54) was involved in the positive regulation of transcription and neuron differentiation, which might participate in neural regeneration [35].

The expression of lncRNAs also showed significant differences between the Sham and the CIPC groups. However, the functions of differentially expressed lncRNAs are yet to be comprehensively studied. Therefore, we studied the functions of the predicted target genes of the differentially expressed lncRNAs based on cis- and trans-acting mechanisms. For example, Trim27 was a putative target of differentially expressed lncRNAs. It manifested protective effects on hepatic, cardiac, and neural injuries [36,37]. Cdip1 was another target mRNA with pro-apoptotic effects [38]. Several studies indicated that the downregulation of Cdip1 improved angiogenesis and attenuated apoptosis after myocardial infarction [39]. However, these target mRNAs always have more than one upstream lncRNA, which complicates the functional analysis. Further experiments are necessary to verify the functions and mechanisms of these compounds.

The biological effects of exosomes are generated from a cluster of molecules, rather than from a single molecule. Therefore, GO and KEGG pathway analyses were performed. Several potentially neuroprotective BP were presented in the top 20 list of the GO analysis of upregulated mRNAs in the CIPC group. These BP included positive regulation of response to external stimulus, regulation of cell growth, regulation of developmental growth, positive regulation of cell projection organization, axonogenesis, and synapse organization. Typical upregulated mRNAs might be potential neuroprotective targets, including Dbn1, Cdkl5, and Wnt3 [[40], [41], [42]]. KEGG pathway analysis demonstrated that several upregulated mRNAs in the CIPC group were enriched in neuroprotective pathways, including the PI3K-Akt signaling pathway, the Hippo signaling pathway, and the Mitophagy-animal pathway. In contrast, there were no downregulated mRNA enriched in the BP with potential neuroprotective effects. However, several mRNAs were enriched in disease-related pathways including Amyotrophic lateral sclerosis, Parkinson's disease, and Legionellosis.

GO and KEGG pathway analyses were also conducted on the target mRNAs of differentially expressed lncRNAs. According to GO analysis, some of the target mRNAs were enriched in BPs with potential neural regenerative functions. For instance, the target mRNAs of upregulated lncRNAs were enriched in axonogenesis [43], and the target mRNAs of downregulated lncRNAs were enriched in the regulation of cell growth, positive regulation of cell cycle, and chromosome segregation [44,45]. According to KEGG pathway analysis, the target mRNAs were also enriched in both injurious and regenerative pathways. The target mRNAs of upregulated lncRNAs were enriched in Lysosome and NF-kappa B signaling pathways, which were related to cell death and inflammation [46,47]. The target mRNAs of downregulated lncRNAs were enriched in Reactive oxygen species and Apoptosis pathways, which were related to the injurious effects after ischemic stroke, and Axon guidance pathway [48], which potentially had neural regenerative effect.

**Fig. 6:**
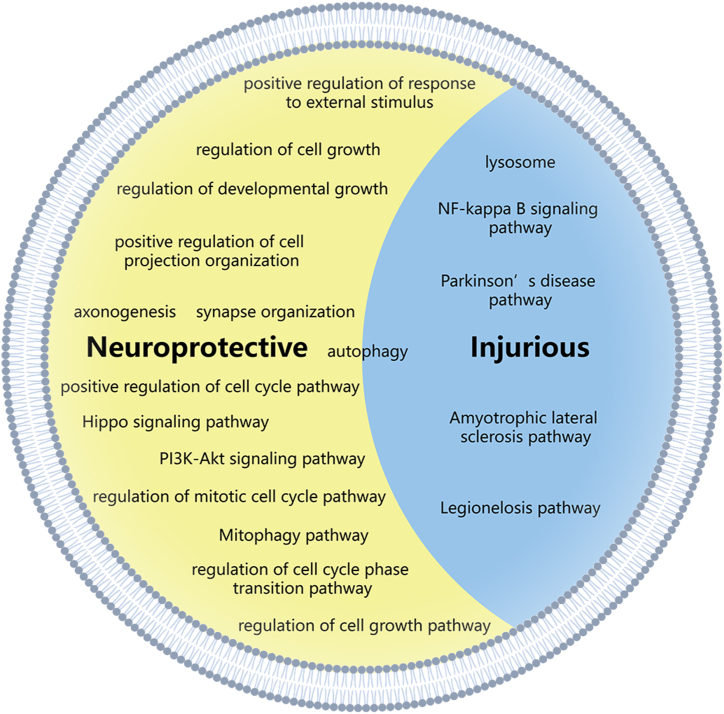
This figure shows the effective components of CIPC-induced exosomes and the directions for future research.

Summarily, the nucleotide expression significantly varied in the exosomes from mice brains after CIPC, and the differentially expressed nucleotides accounted for both neuroprotective and injurious effects. These results seem contradictory, but reasonable in effect. Exosomes eliminate cellular waste, transport signals, and trophic components according to their biological meaning [49,50]. To determine which effect plays a predominant role in CIPC, we performed a preliminary in vivo study with exosomes from the Sham and the CIPC groups. These results indicated that IPCE played a protective role against cellular OGD/R injury. Thus, components with protective or trophic functions against ischemic stroke or cerebral I/R injury may play a major role. Additionally, the results of the in vivo study showed that exosomes from the CIPC group had better neuroprotective effects, indicating that the neuroprotective effects of exosomes were generated from the upregulated components. Thus, our results proved that the upregulated exosome nucleotides after CIPC treatment exerted neuroprotective effects on cerebral I/R injury (Fig. 6).

In this study, we profiled the gene expression differences between exosomes from the CIPC and the Sham groups, preliminarily identifying the potential protective mechanisms of CIPC-induced exosomes. Further investigations are needed to verify our findings and confirm the mechanisms of exosomes. Determining the cellular sources of protective exosomes is a crucial step in investigating the mechanisms of action of exosomes (Fig. 6). Several studies have proposed valuable methods to investigate the cellular sources of tissue-derived exosomes. Liu and colleagues identified the source of exosomes based on the theory that the typical protein markers, or “cluster markers” of each cell type, were highly expressed in their exosomes [51]. Therefore, they calculated the proportion of exosomes derived from each cell cluster based on the proteome analysis data of exosomes and single-cell sequencing data of the source tissue. Santra et al. identified the sources of exosomes using specific biomarkers. For instance, exosomes with high expression of P2Y12 and TMEM119 were regarded as derived from microglia, and exosomes with higher levels of EAAT1 and EAAT2 were regarded as derived from astrocytes [52]. These two methods are practical approaches for investigating the cellular source of brain-derived exosomes and their mechanisms, which deserve further study.

In conclusion, this study provided a landscape of gene expression differences between brain-derived exosomes from the CIPC and the Sham groups. GO and KEGG analyses indicated that many differentially expressed nucleotides were related to neuroprotective BPs or pathways, which was in accordance with our in vivo study, providing a guide for future research. Future studies should focus on illustrating the potential mechanisms of the contents of exosomes and identifying their cellular sources, aiming at finding new therapeutic targets for ischemic stroke and cerebral I/R injury.

## Data availability statement

Data will be made available upon request through e-mail of the corresponding authors.

## CRediT authorship contribution statement

**He Li:** Writing – original draft. **Xiaoxi Zhang:** Writing – review & editing, Methodology. **Hongye Xu:** Methodology. **Hanchen Liu:** Methodology. **Yongxin Zhang:** Methodology, Data curation. **Lei Zhang:** Methodology, Data curation. **Yu Zhou:** Methodology, Data curation. **Yongwei Zhang:** Methodology, Data curation. **Jianmin Liu:** Conceptualization. **Mei Jing:** Writing – review & editing, Conceptualization. **Ping Zhang:** Supervision, Conceptualization. **Pengfei Yang:** Supervision, Conceptualization.

## Declaration of competing interest

The authors declare the following financial interests/personal relationships which may be considered as potential competing interests:Pengfei Yang reports financial support was provided by Stroke Prevention and Treatment Project of the National Health Commission. Pengfei Yang reports financial support was provided by Science and Technology Commission of Shanghai Municipality. He Li reports financial support was provided by Naval Medical University. If there are other authors, they declare that they have no known competing financial interests or personal relationships that could have appeared to influence the work reported in this paper.
