# Supplementary material for: Alternation of gene expression in brain-derived exosomes after cerebral ischemic preconditioning in mice
Source: Heliyon. 2024 Aug 8;10(16):e35936. doi: 10.1016/j.heliyon.2024.e35936 (PMC11367060; doi:10.1016/j.heliyon.2024.e35936)
Supplement: Multimedia component 1 [file mmc1.pdf]

Alternation of gene expression in brain-derived exosomes after cerebral ischemic preconditioning in mice

Supplementary materials

Supplementary Figure 1

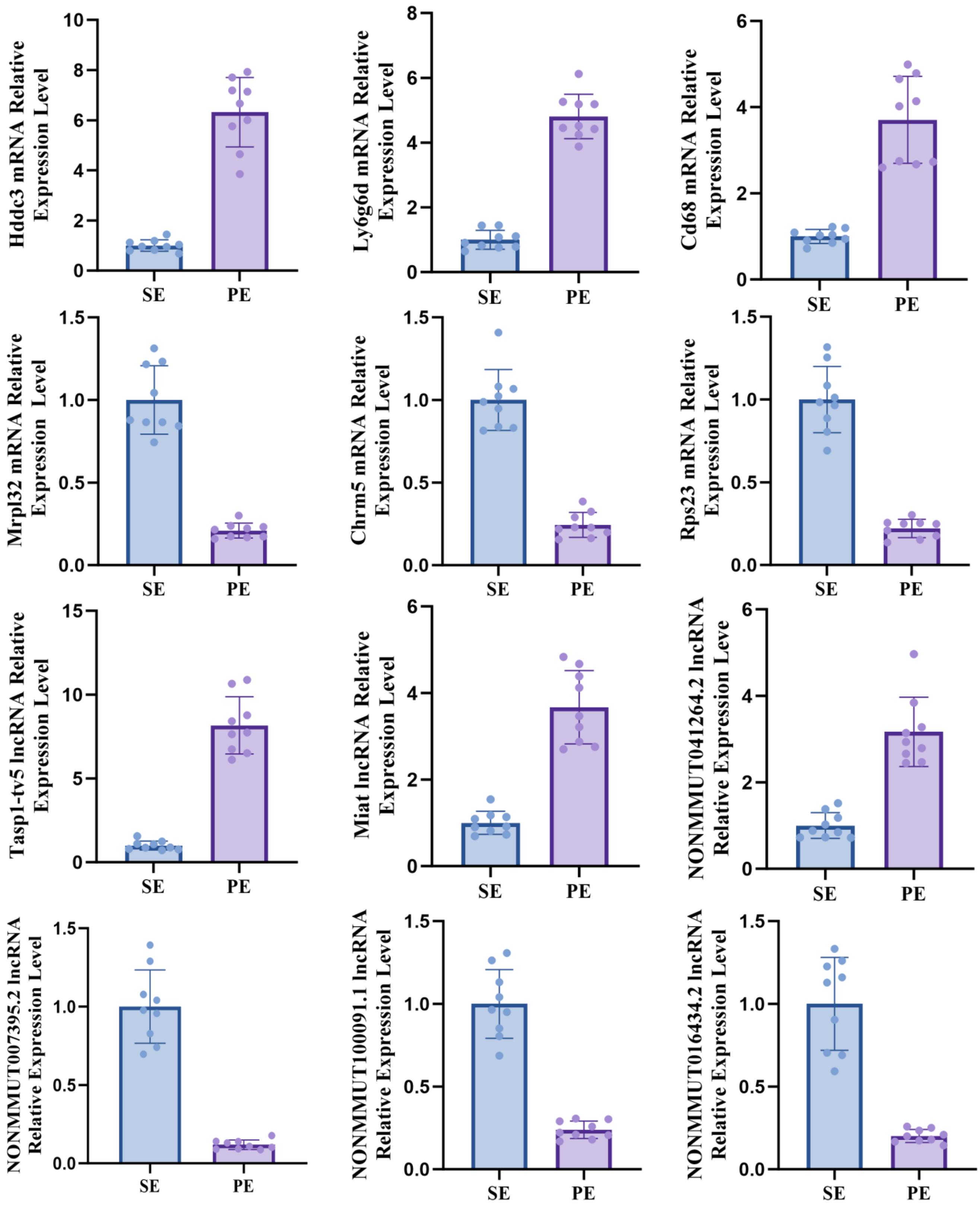

The qRT-PCR showed the relative expression levels of the typical mRNAs and lncRNAs differently expressed in the SE group and the PE group. The primers used in the qRT-PCR were recorded as follows:

Hddc3	Forward	5’-AATCACCCCATCGGTGTAGC-3’
	Reverse	5’-GAACCTGTGCTCCAAAGTGC-3’
Ly6g6d	Forward	5’-GAGCAAGCCAAGCAACCCTC-3’
	Reverse	5’-TCACGTCTCCCACTGACTCT-3’
Cd68	Forward	5’-AGGACCGCTTATAGCCCAAG-3’
	Reverse	5’-TCATCGTGAAGGATGGCAGG-3’
Mrpl32	Forward	5’-CTTTCACTACCATGGCCGGT-3’
	Reverse	5’-CTAATGCTGGTGCCCACGGA-3’
Chrm5	Forward	5’-CAAGCAACTCCTGCCAGATAC-3’
	Reverse	5’-GTGCCGTTGACAGTGGTTTC-3’
Rps23	Forward	5’-CAATGCCTTGTGGGTCCTTCC-3’
	Reverse	5’-ACCACGACACTTGCCCATCT-3’
Tasp-tv5	Forward	5’-GCTGCTCTGGTGGAACTTGA-3’
	Reverse	5’-TTCCACTCAGTGCTCCAACG-3’
Miat	Forward	5’-AAAGGTCACGACTAGCCTGC-3’
	Reverse	5’-AGCTGACCCTTTCTCACACG-3’
NONMMUT041264.2	Forward	5’-AATACTGCCACCCTTGCCTC-3’
	Reverse	5’-CCAGACAGCCTTGTAGCCAA-3’
NONMMUT007395.2	Forward	5’-ACTGTAGCTGTGTGCTCAGG-3’
	Reverse	5’-GCTCACCCAGTGGAAAGTGA-3’
NONMMUT100091.1	Forward	5’-CTTTTGCAAAGGGCGGTCTC-3’
	Reverse	5’-GATGTGCCAAGGCAAGTTGT-3’
NONMMUT016434.2	Forward	5’-TGCCCCCACAGTAGCTATCA-3’
	Reverse	5’-GAAGGGCAGAGATAGAGTGGC-3’

Supplementary Figure 2

A B C

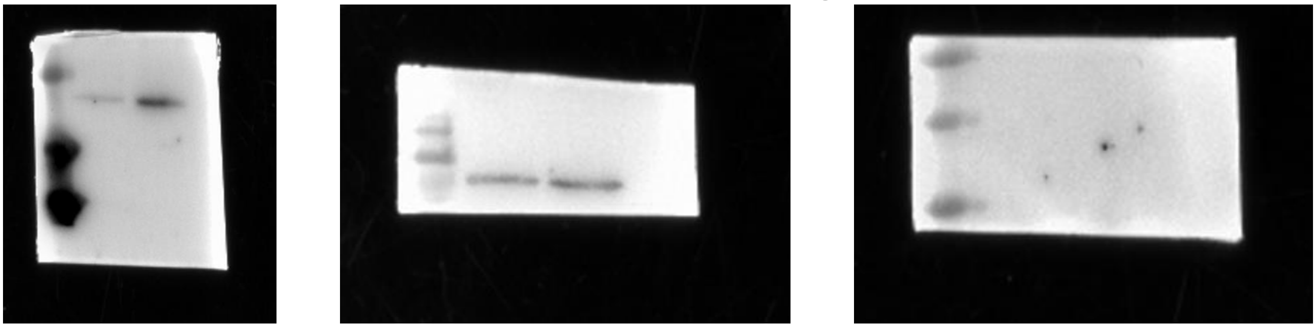

CD63 CD9 Calnexin

The uncropped blots for Figure 1B. Supplementary Figure 2A, 2B, and 2C presented the uncropped blot for CD63, CD9, and Calnexin respectively.

Supplementary Table 1

List of the differentially expressed mRNAs.

mRNA id	mRNA name	CIPC	Sham	log2FC	Pvalue	up-down
ENSMUSG00000036768	Kif15	7.3404	0.0000	Inf	0.0000	UP
ENSMUSG00000092345	Gm20503	18.5280	0.0000	Inf	0.0001	UP
ENSMUSG00000038812	Trmt112	7.5640	0.0000	Inf	0.0001	UP
ENSMUSG00000031861	Lpar2	2.1451	0.0000	Inf	0.0006	UP
ENSMUSG00000086322	E130218I03Rik	5.2180	0.0000	Inf	0.0006	UP
ENSMUSG00000000303	Cdh1	3.5593	0.0000	Inf	0.0012	UP
ENSMUSG00000049539	Hist1h1a	28.9106	0.0000	Inf	0.0024	UP
ENSMUSG00000102881	Gm3807	17.9088	0.0000	Inf	0.0028	UP
ENSMUSG00000099843	Gm7160	9.7167	0.0000	Inf	0.0029	UP
ENSMUSG00000078607	1810010H24Rik	5.2484	0.0000	Inf	0.0029	UP
ENSMUSG00000106676	Gm42895	5.3964	0.0000	Inf	0.0045	UP
ENSMUSG00000025727	A930017K11Rik	5.2603	0.0000	Inf	0.0048	UP
ENSMUSG00000031380	Figf	4.8520	0.0000	Inf	0.0051	UP
ENSMUSG00000085614	1700123M08Rik	6.1325	0.0000	Inf	0.0089	UP
ENSMUSG00000075045	Gm4981	9.4326	0.0000	Inf	0.0095	UP
ENSMUSG00000021624	Cd180	1.6900	0.0000	Inf	0.0095	UP
ENSMUSG00000103651	Gm37206	6.7029	0.0000	Inf	0.0115	UP
ENSMUSG00000089701	Gm15978	12.6530	0.0000	Inf	0.0138	UP
ENSMUSG00000019988	Nedd1	3.7902	0.0000	Inf	0.0149	UP
ENSMUSG00000023902	Zscan10	1.4211	0.0000	Inf	0.0152	UP
ENSMUSG00000078994	Zfp429	3.1971	0.0000	Inf	0.0152	UP
ENSMUSG00000086179	Gm14317	8.0963	0.0000	Inf	0.0181	UP
ENSMUSG00000061991	Hist1h2af	41.3276	0.0000	Inf	0.0182	UP
ENSMUSG00000029343	Crybb1	8.2113	0.0000	Inf	0.0183	UP
ENSMUSG00000050625	Ccdc121	4.6844	0.0000	Inf	0.0184	UP
ENSMUSG00000103522	9430087J23Rik	10.9617	0.0000	Inf	0.0184	UP
ENSMUSG00000097102	2310069G16Rik	14.1497	0.0000	Inf	0.0190	UP
ENSMUSG00000033227	Wnt6	3.9405	0.0000	Inf	0.0192	UP
ENSMUSG00000096953	Gm26571	31.9506	0.0000	Inf	0.0193	UP
ENSMUSG00000033752	Mnd1	17.5053	0.0000	Inf	0.0196	UP
ENSMUSG00000097756	A730056A06Rik	4.8850	0.0000	Inf	0.0207	UP
ENSMUSG00000103301	Gm37804	16.0534	0.0000	Inf	0.0215	UP
ENSMUSG00000034871	Fam151a	8.5224	0.0000	Inf	0.0221	UP
ENSMUSG00000057103	Cml1	5.7899	0.0000	Inf	0.0225	UP
ENSMUSG00000014030	Pax5	0.7575	0.0000	Inf	0.0239	UP
ENSMUSG00000001497	Pax9	1.5476	0.0000	Inf	0.0239	UP
ENSMUSG00000097197	9530027J09Rik	8.5019	0.0000	Inf	0.0240	UP
ENSMUSG00000097502	4930528D03Rik	3.7580	0.0000	Inf	0.0240	UP
ENSMUSG00000084993	Gm12305	15.1461	0.0000	Inf	0.0240	UP
ENSMUSG00000021751	Acox2	2.2608	0.0000	Inf	0.0257	UP
ENSMUSG00000069273	Hist1h3e	14.5198	0.0000	Inf	0.0258	UP
ENSMUSG00000092471	Cyp21a2-ps	8.3080	0.0000	Inf	0.0271	UP
ENSMUSG00000054779	Fgf2os	7.7177	0.0000	Inf	0.0273	UP
ENSMUSG00000028784	Spocd1	3.1297	0.0000	Inf	0.0275	UP
ENSMUSG00000002104	Rapsn	4.4528	0.0000	Inf	0.0285	UP
ENSMUSG00000097706	E030037K01Rik	5.3496	0.0000	Inf	0.0289	UP

ENSMUSG00000096010	Hist4h4	3.0691	0.0000	Inf	0.0299	UP
ENSMUSG00000085498	Gm14023	7.4397	0.0000	Inf	0.0317	UP
ENSMUSG00000058046	4933430I17Rik	6.7065	0.0000	Inf	0.0322	UP
ENSMUSG00000047821	Trim16	1.4431	0.0000	Inf	0.0328	UP
ENSMUSG00000087050	Dhrs13os	5.2312	0.0000	Inf	0.0345	UP
ENSMUSG00000104462	Gm37661	5.9619	0.0000	Inf	0.0347	UP
ENSMUSG00000106063	C030032O16Rik	12.5540	0.0000	Inf	0.0355	UP
ENSMUSG00000040258	Nxph4	5.3622	0.0000	Inf	0.0358	UP
ENSMUSG00000104432	A430027C01Rik	3.8305	0.0000	Inf	0.0359	UP
ENSMUSG00000030708	Dnajb13	3.8993	0.0000	Inf	0.0367	UP
ENSMUSG00000032889	Gm6685	11.0960	0.0000	Inf	0.0380	UP
ENSMUSG00000037921	Ddx60	1.4747	0.0000	Inf	0.0380	UP
ENSMUSG00000034774	Dsg1c	1.8799	0.0000	Inf	0.0382	UP
ENSMUSG00000108227	RP23-226O20.10	9.0403	0.0000	Inf	0.0389	UP
ENSMUSG00000099839	Gm29374	6.3371	0.0000	Inf	0.0391	UP
ENSMUSG00000054763	Defb42	3.4194	0.0000	Inf	0.0418	UP
ENSMUSG00000040340	1700019B03Rik	2.1438	0.0000	Inf	0.0424	UP
ENSMUSG00000030606	Hapln3	1.6012	0.0000	Inf	0.0432	UP
ENSMUSG00000027173	Depdc7	4.3355	0.0000	Inf	0.0436	UP
ENSMUSG00000091091	Kcnmb3	3.8402	0.0000	Inf	0.0441	UP
ENSMUSG00000013766	Ly6g6e	2.9730	0.0000	Inf	0.0444	UP
ENSMUSG00000035364	4930524J08Rik	13.3805	0.0000	Inf	0.0455	UP
ENSMUSG00000044014	Npy5r	2.9065	0.0000	Inf	0.0458	UP
ENSMUSG00000086951	1700051A21Rik	3.4659	0.0000	Inf	0.0464	UP
ENSMUSG00000034768	Asb16	3.2414	0.0000	Inf	0.0470	UP
ENSMUSG00000085051	Gm11542	4.7656	0.0000	Inf	0.0471	UP
ENSMUSG00000056155	Nanos3	8.3612	0.0000	Inf	0.0475	UP
ENSMUSG00000022464	Slc38a4	1.3505	0.0000	Inf	0.0475	UP
ENSMUSG00000076438	Oxct2b	5.2287	0.0000	Inf	0.0478	UP
ENSMUSG00000107606	RP23-411N5.4	2.5501	0.0000	Inf	0.0480	UP
ENSMUSG00000108630	RP24-329F21.2	6.4896	0.0000	Inf	0.0486	UP
ENSMUSG00000032725	Folr2	8.0900	0.0000	Inf	0.0486	UP
ENSMUSG00000091228	Gm20390	12.2592	0.0000	Inf	0.0489	UP
ENSMUSG00000029759	Pon3	1.8263	0.0000	Inf	0.0494	UP
ENSMUSG00000030532	Hddc3	2.7587	0.0394	6.1297	0.0145	UP
ENSMUSG00000073413	Ly6g6d	7.0004	0.1102	5.9892	0.0227	UP
ENSMUSG00000018774	Cd68	7.4227	0.1282	5.8552	0.0090	UP
ENSMUSG00000009487	Otog	1.4386	0.0264	5.7686	0.0446	UP
ENSMUSG00000037406	Htra4	9.5312	0.1815	5.7146	0.0148	UP
ENSMUSG00000024795	Kif20b	2.2635	0.0476	5.5702	0.0069	UP
ENSMUSG00000097643	A130051J06Rik	10.5773	0.2475	5.4176	0.0026	UP
ENSMUSG00000041616	Nppa	15.6973	0.4185	5.2290	0.0440	UP
ENSMUSG00000108035	RP23-251M14.4	3.9206	0.1069	5.1967	0.0374	UP
ENSMUSG00000079489	C030013D06Rik	10.7526	0.3059	5.1356	0.0378	UP
ENSMUSG00000027861	Casq2	2.6323	0.0800	5.0398	0.0371	UP
ENSMUSG00000039099	Wdr93	3.8972	0.1418	4.7809	0.0360	UP
ENSMUSG00000073406	H2-Bl	5.7557	0.2182	4.7214	0.0152	UP
ENSMUSG00000085731	Gm16229	27.9520	1.0761	4.6991	0.0114	UP

ENSMUSG00000038496	Slc19a3	1.9916	0.0781	4.6716	0.0370	UP
ENSMUSG00000070287	Slc35g2	8.3301	0.3345	4.6382	0.0155	UP
ENSMUSG00000020673	Tpo	3.4207	0.1415	4.5950	0.0453	UP
ENSMUSG00000040613	Apobec1	2.7480	0.1332	4.3666	0.0086	UP
ENSMUSG00000097460	Gm26734	10.0373	0.4990	4.3302	0.0263	UP
ENSMUSG00000096056	Gm21986	19.7402	1.0087	4.2906	0.0104	UP
ENSMUSG00000029699	Ssc4d	2.1638	0.1157	4.2248	0.0268	UP
ENSMUSG00000039873	Neurl2	20.5754	1.2781	4.0088	0.0280	UP
ENSMUSG00000024069	Slc30a6	9.0317	0.5794	3.9625	0.0207	UP
ENSMUSG00000063021	Hist1h2ak	44.4565	2.8703	3.9531	0.0209	UP
ENSMUSG00000058743	Kcnj14	8.5830	0.5635	3.9290	0.0334	UP
ENSMUSG00000028214	Gem	5.4242	0.3656	3.8912	0.0262	UP
ENSMUSG00000074235	Gm10649	6.6016	0.5119	3.6889	0.0050	UP
ENSMUSG00000078179	Rnf148	13.2194	1.0700	3.6270	0.0449	UP
ENSMUSG00000102997	Gm17244	5.9400	0.4902	3.5990	0.0306	UP
ENSMUSG00000106464	C130083M11Rik	12.3027	1.0378	3.5674	0.0441	UP
ENSMUSG00000021732	Fgf10	5.7898	0.5138	3.4942	0.0194	UP
ENSMUSG00000035443	Thyn1	38.8501	3.4682	3.4856	0.0014	UP
ENSMUSG00000005677	Nr1i3	2.6303	0.2514	3.3871	0.0309	UP
ENSMUSG00000031379	Pir	3.0177	0.3115	3.2762	0.0460	UP
ENSMUSG00000031465	Angpt2	7.0456	0.7478	3.2361	0.0193	UP
ENSMUSG00000028189	Ctbs	2.8453	0.3078	3.2086	0.0300	UP
ENSMUSG00000051735	Rinl	14.5182	1.5872	3.1933	0.0030	UP
ENSMUSG00000101555	Gm28731	13.6560	1.5681	3.1225	0.0138	UP
ENSMUSG00000030108	Slc6a13	15.2876	1.8923	3.0142	0.0077	UP
ENSMUSG00000074224	4932431P20Rik	1.9708	0.2531	2.9608	0.0406	UP
ENSMUSG00000015668	Pdzd11	7.4059	1.0646	2.7984	0.0303	UP
ENSMUSG00000107498	RP24-257M4.1	8.9802	1.3146	2.7721	0.0462	UP
ENSMUSG00000000125	Wnt3	11.9125	1.7474	2.7692	0.0037	UP
ENSMUSG00000086843	E030013I19Rik	6.2346	0.9198	2.7609	0.0188	UP
ENSMUSG00000105340	Gm42878	15.8609	2.3478	2.7561	0.0261	UP
ENSMUSG00000052395	Rft1	6.5137	1.0069	2.6936	0.0169	UP
ENSMUSG00000051777	Iqcj	26.3399	4.4387	2.5690	0.0199	UP
ENSMUSG00000035378	Shq1	4.4161	0.7589	2.5407	0.0066	UP
ENSMUSG00000034762	Glis1	9.1410	1.6119	2.5036	0.0244	UP
ENSMUSG00000104033	Gm37773	9.8152	1.7813	2.4621	0.0437	UP
ENSMUSG00000031489	Adrb3	5.5354	1.0307	2.4250	0.0157	UP
ENSMUSG00000104245	Gm38155	22.2293	4.1669	2.4154	0.0060	UP
ENSMUSG00000025665	Rps6ka6	4.7492	0.9365	2.3423	0.0221	UP
ENSMUSG00000028862	Map3k6	13.3447	2.7005	2.3050	0.0089	UP
ENSMUSG00000022013	Dnajc15	39.4703	8.2196	2.2636	0.0306	UP
ENSMUSG00000024925	Rnaseh2c	15.7699	3.3709	2.2260	0.0138	UP
ENSMUSG00000017697	Ada	15.8460	3.4553	2.1972	0.0429	UP
ENSMUSG00000019312	Grb7	5.1163	1.1779	2.1189	0.0452	UP
ENSMUSG00000061273	Mmgt1	6.7179	1.5562	2.1100	0.0265	UP
ENSMUSG00000093445	Lrch4	27.8025	6.5482	2.0860	0.0391	UP
ENSMUSG00000032330	Cox7a2	57.6073	13.9737	2.0435	0.0378	UP
ENSMUSG00000102918	Pcdhgc3	50.8523	12.5856	2.0145	0.0003	UP
ENSMUSG00000049555	Tmie	13.6615	3.4249	1.9960	0.0095	UP

ENSMUSG00000071036	Gm10309	27.8124	7.1292	1.9639	0.0275	UP
ENSMUSG00000104378	Gm37510	12.2453	3.1487	1.9594	0.0093	UP
ENSMUSG00000093536	Smim17	12.6991	3.2876	1.9496	0.0274	UP
ENSMUSG00000094568	Smarce1-ps1	25.7826	6.7833	1.9263	0.0456	UP
ENSMUSG00000030752	Kdm8	22.0413	6.0295	1.8701	0.0109	UP
ENSMUSG00000000530	Acvrl1	8.7623	2.3994	1.8686	0.0209	UP
ENSMUSG00000105561	Gm43462	15.4472	4.3814	1.8179	0.0375	UP
ENSMUSG00000100862	Gm10925	234.4265	67.0086	1.8067	0.0002	UP
ENSMUSG00000040740	Slc25a34	18.7332	5.3585	1.8057	0.0479	UP
ENSMUSG00000020890	Gucy2e	6.8281	2.0233	1.7547	0.0264	UP
ENSMUSG00000081058	Hist2h3c2	51.4090	15.2872	1.7497	0.0271	UP
ENSMUSG00000053985	Zfp14	35.4112	10.6235	1.7369	0.0128	UP
ENSMUSG00000099689	Zfp383	9.9317	2.9997	1.7272	0.0409	UP
ENSMUSG00000097431	Gm26782	15.4587	4.6783	1.7243	0.0405	UP
ENSMUSG00000037295	Ldlrap1	7.0761	2.2576	1.6481	0.0458	UP
ENSMUSG00000086968	4933431E20Rik	14.3704	4.5897	1.6466	0.0011	UP
ENSMUSG00000027963	Extl2	7.0618	2.2890	1.6253	0.0162	UP
ENSMUSG00000032035	Ets1	4.5271	1.5379	1.5576	0.0384	UP
ENSMUSG00000041482	Piezo2	3.8233	1.3313	1.5220	0.0447	UP
ENSMUSG00000038668	Lpar1	12.2919	4.3574	1.4962	0.0112	UP
ENSMUSG00000068566	Myadm	5.3695	1.9069	1.4935	0.0190	UP
ENSMUSG00000002249	Tead3	9.1846	3.3328	1.4625	0.0249	UP
ENSMUSG00000030876	Mettl9	106.7778	38.8337	1.4592	0.0002	UP
ENSMUSG00000032182	Yipf2	38.6589	14.2613	1.4387	0.0064	UP
ENSMUSG00000046962	Zbtb21	14.9243	5.5461	1.4281	0.0003	UP
ENSMUSG00000066647	Gm5113	14.5385	5.4588	1.4132	0.0395	UP
ENSMUSG00000034936	Arl4d	72.7685	27.3661	1.4109	0.0090	UP
ENSMUSG00000070953	Rabepk	7.8973	2.9915	1.4005	0.0401	UP
ENSMUSG00000098274	Rpl24	53.1449	20.1712	1.3976	0.0150	UP
ENSMUSG00000021747	4930452B06Rik	43.9755	16.8515	1.3838	0.0269	UP
ENSMUSG00000020541	Tom1l1	6.4837	2.5106	1.3688	0.0443	UP
ENSMUSG00000022360	Atad2	11.4527	4.4353	1.3686	0.0241	UP
ENSMUSG00000021621	Zcchc9	11.3052	4.4118	1.3576	0.0307	UP
ENSMUSG00000028081	Rps3a1	117.4081	46.9969	1.3209	0.0045	UP
ENSMUSG00000033099	Nol12	19.3819	7.8387	1.3060	0.0045	UP
ENSMUSG00000014158	Trpv4	8.4062	3.4627	1.2796	0.0323	UP
ENSMUSG00000038354	Ankrd35	34.4788	14.3755	1.2621	0.0117	UP
ENSMUSG00000037376	Trmt6	15.5705	6.5244	1.2549	0.0040	UP
ENSMUSG00000000088	Cox5a	89.1044	37.3840	1.2531	0.0366	UP
ENSMUSG00000043716	Rpl7	50.2538	21.1876	1.2460	0.0001	UP
ENSMUSG00000029767	Calu	7.9477	3.3632	1.2407	0.0192	UP
ENSMUSG00000030030	1700003E16Rik	41.2259	17.4807	1.2378	0.0082	UP
ENSMUSG00000050663	Trhde	21.4233	9.1593	1.2259	0.0042	UP
ENSMUSG00000032527	Pccb	20.2033	8.7173	1.2126	0.0262	UP
ENSMUSG00000028439	Fam219a	132.5144	57.3700	1.2078	0.0000	UP
ENSMUSG00000021067	Sav1	34.2086	14.8360	1.2053	0.0496	UP
ENSMUSG00000028577	Plaa	13.2260	5.7598	1.1993	0.0134	UP
ENSMUSG00000064138	Fam172a	36.0537	15.7018	1.1992	0.0050	UP
ENSMUSG00000021054	Sgpp1	33.1207	14.4719	1.1945	0.0461	UP

ENSMUSG00000038344	Txlng	14.2481	6.2931	1.1789	0.0012	UP
ENSMUSG00000018861	Fdxr	35.6996	15.7880	1.1771	0.0333	UP
ENSMUSG00000015290	Ubl4a	15.6954	7.0717	1.1502	0.0056	UP
ENSMUSG00000028792	Ak2	39.4442	17.8190	1.1464	0.0169	UP
ENSMUSG00000047371	Zfp768	25.0840	11.8924	1.0767	0.0136	UP
ENSMUSG00000037503	Fam168b	55.0609	26.1440	1.0745	0.0119	UP
ENSMUSG00000007891	Ctsd	66.8945	32.1658	1.0564	0.0176	UP
ENSMUSG00000027297	Ltk	25.5147	12.3594	1.0457	0.0035	UP
ENSMUSG00000064368	mt-Nd6	597.0870	294.8213	1.0181	0.0163	UP
ENSMUSG00000067242	Lgi1	10.1237	5.0254	1.0104	0.0005	UP
ENSMUSG00000036613	Tssc1	80.4522	40.0787	1.0053	0.0140	UP
ENSMUSG00000074247	Dda1	36.5282	18.4400	0.9862	0.0060	UP
ENSMUSG00000064345	mt-Nd2	273.9371	139.1055	0.9777	0.0019	UP
ENSMUSG00000028132	Tmem56	7.2555	3.6893	0.9757	0.0464	UP
ENSMUSG00000002365	Snx9	32.0816	16.5066	0.9587	0.0309	UP
ENSMUSG00000024644	Cndp2	46.9825	24.3343	0.9491	0.0178	UP
ENSMUSG00000025815	Dhtkd1	16.0256	8.3044	0.9484	0.0227	UP
ENSMUSG00000048249	Crebrf	25.6555	13.2983	0.9480	0.0222	UP
ENSMUSG00000022241	Tars	46.4942	24.1821	0.9431	0.0280	UP
ENSMUSG00000028454	Pigo	18.3471	9.5494	0.9421	0.0337	UP
ENSMUSG00000028106	Rprd2	20.8921	10.8898	0.9400	0.0127	UP
ENSMUSG00000032796	Lama1	13.0911	6.8254	0.9396	0.0160	UP
ENSMUSG00000015357	Clpx	16.5659	8.6556	0.9365	0.0442	UP
ENSMUSG00000019970	Sgk1	23.6885	12.4017	0.9337	0.0222	UP
ENSMUSG00000059834	Sclt1	16.9012	8.9737	0.9134	0.0455	UP
ENSMUSG00000044647	Csrnp3	17.5291	9.3442	0.9076	0.0012	UP
ENSMUSG00000021431	Snrnp48	63.8543	34.0695	0.9063	0.0141	UP
ENSMUSG00000038119	Cdon	12.7970	6.8493	0.9018	0.0085	UP
ENSMUSG00000033377	Palmd	17.3092	9.2902	0.8977	0.0143	UP
ENSMUSG00000023460	Rab12	84.6807	45.5102	0.8958	0.0017	UP
ENSMUSG00000028645	Slc2a1	22.5193	12.1049	0.8956	0.0432	UP
ENSMUSG00000034430	Zxdc	15.4025	8.3154	0.8893	0.0050	UP
ENSMUSG00000028277	Ube2j1	38.9612	21.1698	0.8800	0.0355	UP
ENSMUSG00000022635	Zcrb1	32.9025	17.9535	0.8739	0.0414	UP
ENSMUSG00000001156	Mxd1	34.7590	19.0782	0.8655	0.0086	UP
ENSMUSG00000102816	Gm37814	30.9425	17.0004	0.8640	0.0410	UP
ENSMUSG00000039917	Rhbdd2	8.8282	4.8627	0.8604	0.0156	UP
ENSMUSG00000020646	Mboat2	34.8410	19.2254	0.8578	0.0435	UP
ENSMUSG00000040472	Rabggta	34.4010	18.9888	0.8573	0.0232	UP
ENSMUSG00000019892	Lrriq1	16.7756	9.3721	0.8399	0.0215	UP
ENSMUSG00000059939	9430015G10Rik	18.6106	10.4223	0.8365	0.0194	UP
ENSMUSG00000032297	Celf6	18.1674	10.1753	0.8363	0.0292	UP
ENSMUSG00000052301	Doc2a	16.9248	9.5312	0.8284	0.0496	UP
ENSMUSG00000006782	Cnp	84.4534	48.0104	0.8148	0.0215	UP
ENSMUSG00000000134	Tfe3	8.9944	5.1351	0.8086	0.0394	UP
ENSMUSG00000049281	Scn3b	13.4358	7.6816	0.8066	0.0448	UP
ENSMUSG00000020134	Peli1	22.6819	12.9681	0.8066	0.0273	UP
ENSMUSG00000026840	Lamc3	18.0768	10.3814	0.8001	0.0480	UP
ENSMUSG00000073888	Ccl27a	17.2012	9.8798	0.8000	0.0282	UP

ENSMUSG00000033685	Ucp2	16.4075	9.5219	0.7850	0.0397	UP
ENSMUSG00000029504	Ddx51	25.8859	15.0491	0.7825	0.0311	UP
ENSMUSG00000006675	P4htm	47.1936	27.4711	0.7807	0.0114	UP
ENSMUSG00000049225	Pdp1	25.2208	14.7859	0.7704	0.0083	UP
ENSMUSG00000087412	Gm15501	125.9661	74.2075	0.7634	0.0311	UP
ENSMUSG00000032293	Ireb2	30.6424	18.1426	0.7561	0.0411	UP
ENSMUSG00000003527	Dgcr14	57.4911	34.3141	0.7445	0.0156	UP
ENSMUSG00000036403	Cep135	24.3806	14.5832	0.7414	0.0326	UP
ENSMUSG00000024474	Ik	159.2655	95.6504	0.7356	0.0111	UP
ENSMUSG00000031221	Igbp1	19.9250	11.9835	0.7335	0.0440	UP
ENSMUSG00000039536	Stau1	22.2003	13.3882	0.7296	0.0160	UP
ENSMUSG00000025373	Rnf41	68.8319	41.5409	0.7285	0.0278	UP
ENSMUSG00000071379	Hpcal1	107.6026	64.9517	0.7283	0.0386	UP
ENSMUSG00000025616	Usp16	15.3680	9.2805	0.7277	0.0497	UP
ENSMUSG00000041763	Tpp2	11.3208	6.8710	0.7204	0.0261	UP
ENSMUSG00000010936	Vac14	67.1326	40.7551	0.7200	0.0235	UP
ENSMUSG00000027272	Ubr1	29.1875	17.8450	0.7098	0.0098	UP
ENSMUSG00000027303	Ptpra	129.5298	79.2985	0.7079	0.0044	UP
ENSMUSG00000028799	Zfp362	60.1275	36.9638	0.7019	0.0170	UP
ENSMUSG00000086324	Gm15564	93.1367	57.6124	0.6930	0.0467	UP
ENSMUSG00000032131	Abcg4	39.0266	24.1412	0.6930	0.0041	UP
ENSMUSG00000024975	Pdcd4	122.7725	76.2240	0.6877	0.0191	UP
ENSMUSG00000035382	Pcsk7	96.2459	59.8977	0.6842	0.0066	UP
ENSMUSG00000000794	Kcnn3	32.1287	20.0598	0.6796	0.0044	UP
ENSMUSG00000021494	Ddx41	225.8217	141.7906	0.6714	0.0089	UP
ENSMUSG00000028756	Pink1	335.8262	213.3080	0.6548	0.0013	UP
ENSMUSG00000025733	Rhot2	37.5363	23.8844	0.6522	0.0099	UP
ENSMUSG00000004071	Cdip1	29.5064	18.7921	0.6509	0.0288	UP
ENSMUSG00000018909	Arrb1	57.8349	36.9447	0.6466	0.0412	UP
ENSMUSG00000053550	Shisa7	44.5706	28.5508	0.6426	0.0270	UP
ENSMUSG00000037174	Elf2	10.9149	6.9948	0.6420	0.0152	UP
ENSMUSG00000024947	Men1	23.9761	15.4171	0.6371	0.0440	UP
ENSMUSG00000006586	Runx1t1	18.4728	11.9755	0.6253	0.0194	UP
ENSMUSG00000061315	Naca	35.9605	23.4058	0.6195	0.0168	UP
ENSMUSG00000015291	Gdi1	66.4260	43.3413	0.6160	0.0177	UP
ENSMUSG00000053617	Sh3pxd2a	45.4308	29.8577	0.6056	0.0413	UP
ENSMUSG00000030272	Camk1	35.6364	23.4707	0.6025	0.0228	UP
ENSMUSG00000046182	Gsg1l	121.7007	80.2430	0.6009	0.0162	UP
ENSMUSG00000032604	Qars	14.7561	9.7368	0.5998	0.0373	UP
ENSMUSG00000035202	Lars2	7132.6049	4737.8842	0.5902	0.0081	UP
ENSMUSG00000024914	Drap1	54.3242	36.2216	0.5847	0.0361	UP
ENSMUSG00000041607	Mbp	160.6419	107.6106	0.5780	0.0322	UP
ENSMUSG00000042599	Kdm7a	30.1950	20.4029	0.5655	0.0306	UP
ENSMUSG00000064367	mt-Nd5	506.7976	343.5724	0.5608	0.0447	UP
ENSMUSG00000005625	Psmd4	39.5318	26.9795	0.5511	0.0406	UP
ENSMUSG00000019039	Dalrd3	43.1885	29.5136	0.5493	0.0392	UP
ENSMUSG00000035781	R3hdm4	292.8476	201.0727	0.5424	0.0061	UP
ENSMUSG00000013858	Tmem259	48.2890	33.2235	0.5395	0.0414	UP
ENSMUSG00000034021	Pds5b	22.3511	15.4813	0.5298	0.0436	UP

ENSMUSG00000028906	Epb41	40.5349	28.1791	0.5245	0.0216	UP
ENSMUSG00000022536	Glyr1	124.8681	87.2534	0.5171	0.0262	UP
ENSMUSG00000026883	Dab2ip	72.2487	50.6501	0.5124	0.0074	UP
ENSMUSG00000052752	Traf7	24.0601	16.9364	0.5065	0.0261	UP
ENSMUSG00000031292	Cdkl5	35.7144	25.2653	0.4993	0.0379	UP
ENSMUSG00000054013	Tmem179	122.8992	87.5608	0.4891	0.0499	UP
ENSMUSG00000066440	Zfyve26	56.9793	40.8842	0.4789	0.0485	UP
ENSMUSG00000015461	Atf6b	105.1091	75.6168	0.4751	0.0343	UP
ENSMUSG00000055322	Tns1	18.2136	13.2008	0.4644	0.0216	UP
ENSMUSG00000028826	Tmem57	43.7389	31.8050	0.4597	0.0330	UP
ENSMUSG00000022099	Dmtn	217.6810	159.3640	0.4499	0.0198	UP
ENSMUSG00000055866	Per2	105.1098	77.2336	0.4446	0.0475	UP
ENSMUSG00000031386	Hcfc1	48.3058	36.2809	0.4130	0.0361	UP
ENSMUSG00000026425	Srgap2	51.6716	38.8653	0.4109	0.0420	UP
ENSMUSG00000023764	Sfi1	34.4612	25.9637	0.4085	0.0489	UP
ENSMUSG00000034675	Dbn1	503.8862	395.1698	0.3506	0.0343	UP
ENSMUSG00000097786	4933429H19Rik	0.0000	22.3982	-Inf	0.0002	DOWN
ENSMUSG00000031165	Was	0.0000	6.3924	-Inf	0.0006	DOWN
ENSMUSG00000102222	Pcdhga10	0.0000	20.7795	-Inf	0.0008	DOWN
ENSMUSG00000107171	Gm42572	0.0000	4.4611	-Inf	0.0010	DOWN
ENSMUSG00000021636	Marveld2	0.0000	5.3344	-Inf	0.0015	DOWN
ENSMUSG00000026980	Ly75	0.0000	2.0846	-Inf	0.0041	DOWN
ENSMUSG00000024784	Gpha2	0.0000	17.1448	-Inf	0.0045	DOWN
ENSMUSG00000059136	Olfr539	0.0000	14.0389	-Inf	0.0046	DOWN
ENSMUSG00000032717	Mdfi	0.0000	4.1627	-Inf	0.0059	DOWN
ENSMUSG00000063535	Zfp773	0.0000	4.6484	-Inf	0.0068	DOWN
ENSMUSG00000097453	Gm26894	0.0000	21.6945	-Inf	0.0073	DOWN
ENSMUSG00000050150	Slc9b1	0.0000	2.2590	-Inf	0.0075	DOWN
ENSMUSG00000089875	Etohd2	0.0000	4.0494	-Inf	0.0076	DOWN
ENSMUSG00000104687	Gm42899	0.0000	4.7208	-Inf	0.0090	DOWN
ENSMUSG00000097811	2810425M01Rik	0.0000	8.7137	-Inf	0.0101	DOWN
ENSMUSG00000106262	Gm43375	0.0000	6.3359	-Inf	0.0112	DOWN
ENSMUSG00000086949	Gm13066	0.0000	17.2221	-Inf	0.0136	DOWN
ENSMUSG00000063129	Aldoart2	0.0000	5.8580	-Inf	0.0136	DOWN
ENSMUSG00000104040	Gm37563	0.0000	6.2430	-Inf	0.0142	DOWN
ENSMUSG00000103283	Gm37276	0.0000	6.1105	-Inf	0.0149	DOWN
ENSMUSG00000006360	Crip1	0.0000	4.3166	-Inf	0.0161	DOWN
ENSMUSG00000042759	Apobr	0.0000	2.3911	-Inf	0.0181	DOWN
ENSMUSG00000097219	Gm26551	0.0000	3.4989	-Inf	0.0186	DOWN
ENSMUSG00000086801	Gm15943	0.0000	4.6215	-Inf	0.0231	DOWN
ENSMUSG00000039193	Nlrc4	0.0000	3.0155	-Inf	0.0249	DOWN
ENSMUSG00000051456	Hspb3	0.0000	13.8156	-Inf	0.0249	DOWN
ENSMUSG00000097295	Hmgb1-ps8	0.0000	21.0064	-Inf	0.0252	DOWN
ENSMUSG00000074277	Phldb3	0.0000	2.9801	-Inf	0.0259	DOWN
ENSMUSG00000101904	Gm29427	0.0000	9.1108	-Inf	0.0274	DOWN
ENSMUSG00000097125	Gm26885	0.0000	13.7221	-Inf	0.0292	DOWN
ENSMUSG00000075558	Gm17151	0.0000	30.1041	-Inf	0.0294	DOWN
ENSMUSG00000108170	RP23-92M3.4	0.0000	11.4247	-Inf	0.0298	DOWN
ENSMUSG00000087439	Gm15788	0.0000	5.3332	-Inf	0.0299	DOWN

ENSMUSG00000102863	Gm37639	0.0000	4.7780	-Inf	0.0307	DOWN
ENSMUSG00000105082	Aqp10-ps	0.0000	12.9575	-Inf	0.0312	DOWN
ENSMUSG00000007033	Hspa1l	0.0000	6.1926	-Inf	0.0313	DOWN
ENSMUSG00000105827	Hist2h2bb	0.0000	30.0684	-Inf	0.0319	DOWN
ENSMUSG00000089975	Gm7420	0.0000	13.5338	-Inf	0.0320	DOWN
ENSMUSG00000048521	Cxcr6	0.0000	3.4314	-Inf	0.0327	DOWN
ENSMUSG00000038422	Hdhd3	0.0000	6.3059	-Inf	0.0335	DOWN
ENSMUSG00000041736	Tspo	0.0000	12.5389	-Inf	0.0368	DOWN
ENSMUSG00000087302	Sox5os1	0.0000	4.5079	-Inf	0.0372	DOWN
ENSMUSG00000033080	Vsx1	0.0000	2.6557	-Inf	0.0373	DOWN
ENSMUSG00000051262	Cml3	0.0000	1.8316	-Inf	0.0387	DOWN
ENSMUSG00000105935	Gm43628	0.0000	7.3531	-Inf	0.0388	DOWN
ENSMUSG00000104925	Gm43061	0.0000	5.2877	-Inf	0.0389	DOWN
ENSMUSG00000105637	Gm42480	0.0000	2.8353	-Inf	0.0390	DOWN
ENSMUSG00000106522	Gm42871	0.0000	27.8954	-Inf	0.0398	DOWN
ENSMUSG00000107620	RP23-449F12.5	0.0000	32.9819	-Inf	0.0404	DOWN
ENSMUSG00000096990	Gm26790	0.0000	5.8966	-Inf	0.0409	DOWN
ENSMUSG00000079711	Smok4a	0.0000	2.3156	-Inf	0.0411	DOWN
ENSMUSG00000095970	Gm19402	0.0000	11.6565	-Inf	0.0447	DOWN
ENSMUSG00000085847	Gm14540	0.0000	3.8221	-Inf	0.0450	DOWN
ENSMUSG00000096904	Gm3591	0.0000	4.0639	-Inf	0.0450	DOWN
ENSMUSG00000020017	Hal	0.0000	1.2286	-Inf	0.0468	DOWN
ENSMUSG00000102828	Gm38182	0.0000	5.4239	-Inf	0.0473	DOWN
ENSMUSG00000074603	Gm10729	0.0000	2.8091	-Inf	0.0489	DOWN
ENSMUSG00000106025	Gm42940	0.0000	4.8540	-Inf	0.0499	DOWN
ENSMUSG00000097722	Gm26841	0.1512	12.2278	-6.3378	0.0110	DOWN
ENSMUSG00000104061	Gm37953	0.1434	10.5375	-6.1994	0.0061	DOWN
ENSMUSG00000015672	Mrpl32	0.3518	24.1017	-6.0984	0.0026	DOWN
ENSMUSG00000085337	Gm15964	0.0984	6.2016	-5.9776	0.0097	DOWN
ENSMUSG00000104406	Gm38014	0.1034	6.2562	-5.9186	0.0295	DOWN
ENSMUSG00000108388	RP23-172P1.1	0.2712	14.4066	-5.7311	0.0160	DOWN
ENSMUSG00000074939	Chrm5	0.1689	8.8191	-5.7060	0.0469	DOWN
ENSMUSG00000105198	Gm42502	0.2481	12.2135	-5.6216	0.0437	DOWN
ENSMUSG00000104116	Gm37296	0.1472	6.3868	-5.4391	0.0072	DOWN
ENSMUSG00000049517	Rps23	0.3573	15.4884	-5.4378	0.0307	DOWN
ENSMUSG00000102147	Gm38055	0.1940	7.9155	-5.3503	0.0189	DOWN
ENSMUSG00000027401	Tgm3	0.0653	2.5856	-5.3066	0.0325	DOWN
ENSMUSG00000107103	Gm43164	0.1269	4.9420	-5.2828	0.0407	DOWN
ENSMUSG00000106043	Gm42968	0.1969	7.4143	-5.2348	0.0404	DOWN
ENSMUSG00000106432	Gm43259	0.1142	4.2899	-5.2313	0.0071	DOWN
ENSMUSG00000107276	Gm42858	0.2196	8.0229	-5.1910	0.0141	DOWN
ENSMUSG00000056328	Myh1	0.0751	2.6112	-5.1203	0.0392	DOWN
ENSMUSG00000104416	Gm37067	0.3958	13.5504	-5.0974	0.0150	DOWN
ENSMUSG00000024131	Slc3a1	0.9818	31.7970	-5.0173	0.0162	DOWN
ENSMUSG00000075053	Vdac3-ps1	0.4147	13.2737	-5.0003	0.0184	DOWN
ENSMUSG00000107482	RP23-74K24.2	0.4502	14.1394	-4.9729	0.0228	DOWN
ENSMUSG00000026175	Vil1	0.1710	5.1713	-4.9187	0.0211	DOWN
ENSMUSG00000079444	Gm21981	0.1375	4.0143	-4.8672	0.0388	DOWN
ENSMUSG00000096991	Gm26789	0.2993	7.3953	-4.6269	0.0064	DOWN

ENSMUSG00000047591	Mafa	0.8468	20.9192	-4.6267	0.0048	DOWN
ENSMUSG00000042793	Lgr6	0.2579	6.3151	-4.6137	0.0000	DOWN
ENSMUSG00000063529	Stmnd1	0.5478	12.6690	-4.5315	0.0204	DOWN
ENSMUSG00000066042	Med18	0.3255	7.3343	-4.4941	0.0421	DOWN
ENSMUSG00000086536	Gm12264	1.4978	31.7217	-4.4046	0.0123	DOWN
ENSMUSG00000018239	Zcchc10	0.5735	11.9482	-4.3809	0.0148	DOWN
ENSMUSG00000103477	5930409G06Rik	0.3499	6.5814	-4.2332	0.0420	DOWN
ENSMUSG00000027997	Casp6	0.2734	4.9540	-4.1797	0.0079	DOWN
ENSMUSG00000075271	Ttc30a1	0.4025	6.6950	-4.0559	0.0258	DOWN
ENSMUSG00000057092	Fxyd3	0.1870	3.1012	-4.0518	0.0384	DOWN
ENSMUSG00000100691	2010320M18Rik	1.2768	20.9195	-4.0343	0.0260	DOWN
ENSMUSG00000089984	Fbxo24	0.2232	3.5840	-4.0052	0.0395	DOWN
ENSMUSG00000032041	Tirap	0.2642	4.2157	-3.9960	0.0008	DOWN
ENSMUSG00000035704	Alg8	0.3576	5.5453	-3.9549	0.0296	DOWN
ENSMUSG00000104391	Gm37960	0.6245	8.8212	-3.8202	0.0329	DOWN
ENSMUSG00000104118	Gm37298	0.5302	7.2827	-3.7799	0.0195	DOWN
ENSMUSG00000068196	Col8a1	0.5864	7.5300	-3.6826	0.0119	DOWN
ENSMUSG00000028100	Nudt17	0.4214	5.3088	-3.6552	0.0250	DOWN
ENSMUSG00000103529	A730089K16Rik	1.1717	14.3798	-3.6173	0.0125	DOWN
ENSMUSG00000030647	Ndufc2	1.2628	15.3450	-3.6031	0.0393	DOWN
ENSMUSG00000086075	Gm15728	0.3989	4.8322	-3.5987	0.0358	DOWN
ENSMUSG00000102813	Gm37795	0.7689	8.9754	-3.5451	0.0411	DOWN
ENSMUSG00000029595	Lhx5	0.7182	8.3623	-3.5414	0.0254	DOWN
ENSMUSG00000047832	Cdca4	0.7121	8.2831	-3.5401	0.0375	DOWN
ENSMUSG00000025213	Kazald1	1.4668	15.2953	-3.3824	0.0166	DOWN
ENSMUSG00000032419	Tbx18	0.6235	6.1634	-3.3052	0.0144	DOWN
ENSMUSG00000105981	2810428J06Rik	0.5556	5.3986	-3.2806	0.0499	DOWN
ENSMUSG00000106940	Gm42930	1.1930	11.2156	-3.2328	0.0038	DOWN
ENSMUSG00000053914	Kdm4d	0.6739	5.9380	-3.1394	0.0189	DOWN
ENSMUSG00000055653	Gpc3	0.5201	4.4766	-3.1056	0.0488	DOWN
ENSMUSG00000091736	Yy2	1.9139	15.7162	-3.0376	0.0198	DOWN
ENSMUSG00000048581	E130311K13Rik	0.7457	5.9270	-2.9906	0.0282	DOWN
ENSMUSG00000029072	Tas1r3	3.3315	26.3896	-2.9857	0.0000	DOWN
ENSMUSG00000104753	Gm43253	2.1412	16.8579	-2.9769	0.0169	DOWN
ENSMUSG00000028889	Yrdc	2.8841	22.6715	-2.9747	0.0021	DOWN
ENSMUSG00000086441	Gm15046	8.5604	66.6088	-2.9600	0.0276	DOWN
ENSMUSG00000064357	mt-Atp6	13.9911	99.7460	-2.8337	0.0151	DOWN
ENSMUSG00000028801	Stpg1	0.8956	6.2393	-2.8005	0.0325	DOWN
ENSMUSG00000097462	9530026P05Rik	0.4001	2.7072	-2.7583	0.0365	DOWN
ENSMUSG00000104420	E230020A03Rik	1.9752	13.1443	-2.7343	0.0312	DOWN
ENSMUSG00000021606	Ndufs6	7.3571	48.8214	-2.7303	0.0047	DOWN
ENSMUSG00000102702	Gm37260	3.5347	23.3258	-2.7223	0.0132	DOWN
ENSMUSG00000104851	E030026E10Rik	1.9415	12.3455	-2.6687	0.0444	DOWN
ENSMUSG00000086363	A330102I10Rik	2.7906	17.4699	-2.6462	0.0132	DOWN
ENSMUSG00000053263	Gm12592	3.1996	19.8637	-2.6342	0.0056	DOWN
ENSMUSG00000097026	Gm26786	2.5688	15.5184	-2.5948	0.0015	DOWN
ENSMUSG00000006720	Zfp184	0.5584	3.1693	-2.5048	0.0313	DOWN
ENSMUSG00000044528	Tram1l1	1.8193	10.1978	-2.4868	0.0291	DOWN
ENSMUSG00000107201	5930420M18Rik	2.8417	15.8914	-2.4834	0.0280	DOWN

ENSMUSG00000037664	Cdkn1c	2.6073	14.5477	-2.4802	0.0148	DOWN
ENSMUSG00000103207	Gm9874	5.7083	30.3479	-2.4105	0.0086	DOWN
ENSMUSG00000102737	1700016A09Rik	6.9018	36.6581	-2.4091	0.0358	DOWN
ENSMUSG00000055547	Apobec4	5.1546	27.3725	-2.4088	0.0159	DOWN
ENSMUSG00000028688	Toe1	1.2371	6.2728	-2.3421	0.0146	DOWN
ENSMUSG00000029209	Gnpda2	0.7574	3.7453	-2.3059	0.0322	DOWN
ENSMUSG00000049160	4930469G21Rik	2.7587	13.3985	-2.2800	0.0477	DOWN
ENSMUSG00000083111	Gm14421	5.5827	26.8060	-2.2635	0.0124	DOWN
ENSMUSG00000097532	Gm4349	1.8955	8.8505	-2.2231	0.0449	DOWN
ENSMUSG00000106691	2700029L08Rik	7.5761	33.1088	-2.1277	0.0437	DOWN
ENSMUSG00000097518	Gm26694	12.8175	55.4667	-2.1135	0.0051	DOWN
ENSMUSG00000020411	Nipal4	2.8862	12.2290	-2.0831	0.0461	DOWN
ENSMUSG00000097331	F420014N23Rik	3.8914	16.3847	-2.0740	0.0491	DOWN
ENSMUSG00000026005	Rpe	4.9486	20.6412	-2.0604	0.0021	DOWN
ENSMUSG00000097944	A130014A01Rik	4.8125	19.4698	-2.0164	0.0200	DOWN
ENSMUSG00000029366	Dck	2.6347	10.6313	-2.0126	0.0458	DOWN
ENSMUSG00000097411	B430218F22Rik	8.7457	34.7969	-1.9923	0.0195	DOWN
ENSMUSG00000031770	Herpud1	4.8438	18.7935	-1.9560	0.0147	DOWN
ENSMUSG00000022747	St3gal6	1.9075	7.2747	-1.9312	0.0144	DOWN
ENSMUSG00000073906	Olfr692	9.4522	35.6099	-1.9136	0.0299	DOWN
ENSMUSG00000070392	Gm20634	10.1569	37.9470	-1.9015	0.0000	DOWN
ENSMUSG00000047844	Bex4	17.5626	64.6545	-1.8802	0.0335	DOWN
ENSMUSG00000070436	Serpinh1	8.5238	31.0044	-1.8629	0.0102	DOWN
ENSMUSG00000031907	Zfp90	9.6073	34.8027	-1.8570	0.0244	DOWN
ENSMUSG00000064220	Hist2h2aa1	60.3957	213.1182	-1.8191	0.0238	DOWN
ENSMUSG00000086484	Nron	1.1815	4.1492	-1.8122	0.0407	DOWN
ENSMUSG00000103364	Gm38157	8.3373	29.2634	-1.8114	0.0159	DOWN
ENSMUSG00000051008	4930412M03Rik	17.2508	60.2519	-1.8043	0.0098	DOWN
ENSMUSG00000097090	Gm26724	8.2858	28.8684	-1.8008	0.0239	DOWN
ENSMUSG00000070469	Adamtsl3	1.5992	5.5014	-1.7824	0.0388	DOWN
ENSMUSG00000039725	Trp53rka	13.9125	47.6759	-1.7769	0.0031	DOWN
ENSMUSG00000021764	Ndufs4	16.0754	54.0384	-1.7491	0.0160	DOWN
ENSMUSG00000026788	Zbtb43	3.7826	12.6986	-1.7472	0.0029	DOWN
ENSMUSG00000075027	4631405J19Rik	2.5316	8.4006	-1.7304	0.0419	DOWN
ENSMUSG00000103085	Gm38120	4.6166	14.8391	-1.6845	0.0178	DOWN
ENSMUSG00000061374	Fiz1	6.8673	21.9875	-1.6789	0.0225	DOWN
ENSMUSG00000021671	Poc5	10.9921	35.1006	-1.6750	0.0153	DOWN
ENSMUSG00000027203	Dut	8.1795	25.8430	-1.6597	0.0041	DOWN
ENSMUSG00000093553	Gm20633	15.9176	50.2795	-1.6593	0.0062	DOWN
ENSMUSG00000021541	Trpc7	2.4696	7.7687	-1.6534	0.0260	DOWN
ENSMUSG00000059183	Mtfmt	6.2329	18.8468	-1.5963	0.0260	DOWN
ENSMUSG00000104868	Gm9954	11.4522	34.1352	-1.5756	0.0033	DOWN
ENSMUSG00000091537	Tma7	10.3638	30.7226	-1.5677	0.0341	DOWN
ENSMUSG00000085609	1700016P03Rik	19.0029	56.1223	-1.5624	0.0313	DOWN
ENSMUSG00000049871	Nlrc3	1.3789	3.9852	-1.5312	0.0319	DOWN
ENSMUSG00000073073	Gm8098	21.3295	61.0772	-1.5178	0.0096	DOWN
ENSMUSG00000039556	Ppp1r3f	6.4942	18.4538	-1.5067	0.0039	DOWN
ENSMUSG00000026174	Rqcd1	12.2838	34.1500	-1.4751	0.0046	DOWN
ENSMUSG00000040759	Cmtm5	24.9142	68.0410	-1.4494	0.0382	DOWN

ENSMUSG00000020063	Sirt1	2.7352	7.4315	-1.4420	0.0105	DOWN
ENSMUSG00000028114	Mettl14	2.8537	7.5665	-1.4068	0.0131	DOWN
ENSMUSG00000043207	Zmpste24	2.6420	7.0030	-1.4063	0.0413	DOWN
ENSMUSG00000026479	Lamc2	3.7235	9.8012	-1.3963	0.0334	DOWN
ENSMUSG00000031608	Galnt7	3.7710	9.8918	-1.3913	0.0266	DOWN
ENSMUSG00000039294	BC017643	5.8398	15.2253	-1.3825	0.0265	DOWN
ENSMUSG00000101823	Gm29438	12.4312	32.2651	-1.3760	0.0327	DOWN
ENSMUSG00000034684	Sema3f	6.2855	16.2012	-1.3660	0.0051	DOWN
ENSMUSG00000021831	Ero1l	8.2876	21.1987	-1.3550	0.0399	DOWN
ENSMUSG00000062646	Ganc	1.7529	4.4078	-1.3303	0.0437	DOWN
ENSMUSG00000056305	Usp39	15.7492	39.4051	-1.3231	0.0270	DOWN
ENSMUSG00000074405	Zfp865	27.3523	67.4254	-1.3016	0.0320	DOWN
ENSMUSG00000097220	Gm26599	9.6073	23.6291	-1.2984	0.0389	DOWN
ENSMUSG00000038175	Mylip	11.1885	27.4752	-1.2961	0.0074	DOWN
ENSMUSG00000074358	Ccdc61	13.7256	33.1081	-1.2703	0.0215	DOWN
ENSMUSG00000067594	Krt77	14.9298	35.9357	-1.2672	0.0182	DOWN
ENSMUSG00000041028	Ghitm	37.2908	89.6752	-1.2659	0.0332	DOWN
ENSMUSG00000024055	Cyp4f13	5.4269	12.9338	-1.2530	0.0484	DOWN
ENSMUSG00000086924	Gm11766	18.1685	43.1411	-1.2476	0.0117	DOWN
ENSMUSG00000064120	Mocs1	4.5573	10.8199	-1.2474	0.0419	DOWN
ENSMUSG00000038094	Atp13a4	4.3298	10.2574	-1.2443	0.0267	DOWN
ENSMUSG00000074738	B930041F14Rik	16.5017	38.7140	-1.2302	0.0304	DOWN
ENSMUSG00000025236	Adpgk	25.2318	58.9932	-1.2253	0.0093	DOWN
ENSMUSG00000031902	Nfatc3	7.1041	16.5572	-1.2207	0.0396	DOWN
ENSMUSG00000034009	Rxfp1	3.8462	8.9127	-1.2124	0.0195	DOWN
ENSMUSG00000056947	Mab21l1	24.8669	57.4826	-1.2089	0.0304	DOWN
ENSMUSG00000103436	Gm36995	13.0576	29.9412	-1.1972	0.0110	DOWN
ENSMUSG00000036275	9530068E07Rik	6.8088	15.5893	-1.1951	0.0364	DOWN
ENSMUSG00000005846	Rsl1d1	13.4734	30.7380	-1.1899	0.0257	DOWN
ENSMUSG00000000959	Oxa1l	16.1163	36.4872	-1.1789	0.0174	DOWN
ENSMUSG00000095159	Tubb4b-ps1	27.1582	61.0977	-1.1697	0.0345	DOWN
ENSMUSG00000049878	Rlf	5.7503	12.9313	-1.1692	0.0207	DOWN
ENSMUSG00000016510	Mtif3	5.9008	13.2376	-1.1657	0.0192	DOWN
ENSMUSG00000054871	Tmem158	36.2184	81.1969	-1.1647	0.0090	DOWN
ENSMUSG00000062526	Mppe1	16.0750	35.8587	-1.1575	0.0343	DOWN
ENSMUSG00000056116	H2-T22	7.7334	17.1820	-1.1517	0.0309	DOWN
ENSMUSG00000079614	Seh1l	12.1545	26.9775	-1.1503	0.0124	DOWN
ENSMUSG00000050846	Zfp623	47.2562	104.6788	-1.1474	0.0011	DOWN
ENSMUSG00000008348	Ubc	21.3650	47.3226	-1.1473	0.0046	DOWN
ENSMUSG00000061524	Zic2	18.2742	40.0887	-1.1334	0.0192	DOWN
ENSMUSG00000025133	Ints4	7.5013	16.3795	-1.1267	0.0048	DOWN
ENSMUSG00000078202	Nrarp	14.5640	31.7538	-1.1245	0.0429	DOWN
ENSMUSG00000006403	Adamts4	9.9116	21.5915	-1.1233	0.0163	DOWN
ENSMUSG00000028995	Fam126a	4.7278	10.2554	-1.1171	0.0495	DOWN
ENSMUSG00000063894	Zkscan8	8.8386	19.1124	-1.1126	0.0416	DOWN
ENSMUSG00000045216	Hs6st1	24.2770	52.3763	-1.1093	0.0071	DOWN
ENSMUSG00000056708	Ier5	42.8395	92.3606	-1.1083	0.0023	DOWN
ENSMUSG00000042202	Slc35e2	8.1392	17.5321	-1.1070	0.0136	DOWN
ENSMUSG00000090115	Usp49	8.0484	17.1335	-1.0900	0.0391	DOWN

ENSMUSG00000039509	Nup133	13.9632	29.3726	-1.0728	0.0170	DOWN
ENSMUSG00000030095	Tmem43	9.2977	19.5327	-1.0709	0.0468	DOWN
ENSMUSG00000033793	Atp6v1h	5.4799	11.4652	-1.0650	0.0063	DOWN
ENSMUSG00000074748	Atxn7l3b	53.0066	109.9689	-1.0529	0.0011	DOWN
ENSMUSG00000027840	Wnt2b	7.7490	16.0262	-1.0484	0.0459	DOWN
ENSMUSG00000038416	Cdc16	11.9278	24.1421	-1.0172	0.0498	DOWN
ENSMUSG00000064105	Cnnm2	40.5130	81.7725	-1.0132	0.0296	DOWN
ENSMUSG00000036752	Tubb4b	41.1690	83.0913	-1.0131	0.0046	DOWN
ENSMUSG00000040312	Cchcr1	11.2826	22.7293	-1.0105	0.0199	DOWN
ENSMUSG00000032172	Olfm2	42.4480	85.4912	-1.0101	0.0401	DOWN
ENSMUSG00000038174	Fam126b	3.8319	7.7124	-1.0091	0.0286	DOWN
ENSMUSG00000021326	Trim27	10.9248	21.8866	-1.0024	0.0227	DOWN
ENSMUSG00000032786	Alas1	14.6761	29.2536	-0.9951	0.0331	DOWN
ENSMUSG00000024325	Ring1	30.6482	60.7761	-0.9877	0.0039	DOWN
ENSMUSG00000027330	Cdc25b	13.6939	27.1507	-0.9875	0.0069	DOWN
ENSMUSG00000020463	Smek2	13.2593	26.2344	-0.9845	0.0400	DOWN
ENSMUSG00000018008	Cyth4	15.4093	30.3170	-0.9763	0.0412	DOWN
ENSMUSG00000022016	Akap11	40.5756	79.2469	-0.9657	0.0028	DOWN
ENSMUSG00000078515	Ddi2	21.8837	42.7116	-0.9648	0.0023	DOWN
ENSMUSG00000072915	Gm12258	11.0127	21.3317	-0.9538	0.0400	DOWN
ENSMUSG00000069020	Urm1	15.1003	29.1382	-0.9483	0.0374	DOWN
ENSMUSG00000047361	Gm973	11.8655	22.8498	-0.9454	0.0227	DOWN
ENSMUSG00000018428	Akap1	27.9773	53.6926	-0.9405	0.0131	DOWN
ENSMUSG00000005374	Tbl2	6.3135	12.0853	-0.9367	0.0500	DOWN
ENSMUSG00000033124	Atg9a	15.4793	29.5669	-0.9336	0.0468	DOWN
ENSMUSG00000033467	Crlf2	24.6893	46.9796	-0.9281	0.0270	DOWN
ENSMUSG00000025352	Gdf11	23.8908	45.4394	-0.9275	0.0456	DOWN
ENSMUSG00000048696	Mex3d	13.3097	25.2571	-0.9242	0.0118	DOWN
ENSMUSG00000028771	Ptpn12	13.6824	25.9610	-0.9240	0.0131	DOWN
ENSMUSG00000028410	Dnaja1	3.5395	6.7114	-0.9230	0.0481	DOWN
ENSMUSG00000030704	Rab6a	47.3746	89.4020	-0.9162	0.0064	DOWN
ENSMUSG00000032434	Cmtm6	20.3116	38.2504	-0.9132	0.0402	DOWN
ENSMUSG00000000439	Mkrn2	9.8244	18.4543	-0.9095	0.0458	DOWN
ENSMUSG00000031672	Got2	32.5645	60.9031	-0.9032	0.0468	DOWN
ENSMUSG00000007987	Ift22	15.6266	29.1657	-0.9003	0.0448	DOWN
ENSMUSG00000016619	Nup50	22.3765	41.7612	-0.9002	0.0341	DOWN
ENSMUSG00000059866	Tnip2	21.0425	39.1113	-0.8943	0.0173	DOWN
ENSMUSG00000020175	Rab36	11.6629	21.6391	-0.8917	0.0083	DOWN
ENSMUSG00000003382	Etv3	4.1663	7.6992	-0.8860	0.0412	DOWN
ENSMUSG00000024831	Ighmbp2	13.3656	24.6644	-0.8839	0.0205	DOWN
ENSMUSG00000108585	Nova2	205.3694	374.9670	-0.8685	0.0305	DOWN
ENSMUSG00000040451	Sgms1	8.4349	15.3865	-0.8672	0.0032	DOWN
ENSMUSG00000048076	Arf1	55.4566	101.0332	-0.8654	0.0380	DOWN
ENSMUSG00000039354	Smarcal1	27.1498	49.3051	-0.8608	0.0028	DOWN
ENSMUSG00000067873	Htatsf1	43.4798	78.8830	-0.8594	0.0037	DOWN
ENSMUSG00000003581	Rnf215	35.9677	65.1749	-0.8576	0.0254	DOWN
ENSMUSG00000036957	Lrfn3	37.3424	67.5017	-0.8541	0.0435	DOWN
ENSMUSG00000037275	Gemin5	13.1272	23.6366	-0.8485	0.0142	DOWN
ENSMUSG00000025138	Sirt7	12.1779	21.5668	-0.8245	0.0102	DOWN

ENSMUSG00000030256	Bhlhe41	28.1999	49.5247	-0.8125	0.0347	DOWN
ENSMUSG00000078786	BC024978	12.2004	21.3866	-0.8098	0.0448	DOWN
ENSMUSG00000027198	Ext2	13.9289	24.3853	-0.8079	0.0234	DOWN
ENSMUSG00000024565	Sall3	13.9499	24.4119	-0.8073	0.0465	DOWN
ENSMUSG00000011257	Pabpc4	11.6975	20.4653	-0.8070	0.0240	DOWN
ENSMUSG00000020358	Hnrnpab	60.5192	105.6338	-0.8036	0.0029	DOWN
ENSMUSG00000104475	D630036G22Rik	35.8651	62.4451	-0.8000	0.0352	DOWN
ENSMUSG00000036545	Adamts2	8.4643	14.7329	-0.7996	0.0438	DOWN
ENSMUSG00000053552	Ebf4	36.2741	63.0258	-0.7970	0.0089	DOWN
ENSMUSG00000033006	Sox10	69.5598	120.5925	-0.7938	0.0034	DOWN
ENSMUSG00000016921	Srsf6	31.5400	54.5812	-0.7912	0.0023	DOWN
ENSMUSG00000003344	Btbd2	33.3231	57.6596	-0.7910	0.0183	DOWN
ENSMUSG00000049643	2310022A10Rik	11.3847	19.4502	-0.7727	0.0472	DOWN
ENSMUSG00000008206	Cers4	12.4689	21.1874	-0.7649	0.0244	DOWN
ENSMUSG00000032010	Usp2	19.4582	32.8745	-0.7566	0.0159	DOWN
ENSMUSG00000041124	Msantd4	41.9627	70.4841	-0.7482	0.0287	DOWN
ENSMUSG00000048833	Slc39a9	21.5845	36.2361	-0.7474	0.0276	DOWN
ENSMUSG00000024590	Lmnb1	35.7334	59.6062	-0.7382	0.0484	DOWN
ENSMUSG00000029151	Slc30a3	34.0158	56.1867	-0.7240	0.0335	DOWN
ENSMUSG00000032115	Hyou1	21.0850	34.5001	-0.7104	0.0216	DOWN
ENSMUSG00000025316	Banp	21.5319	35.0588	-0.7033	0.0239	DOWN
ENSMUSG00000078578	Ube2d3	4.4911	7.3007	-0.7010	0.0161	DOWN
ENSMUSG00000058440	Nrf1	8.1488	13.0504	-0.6794	0.0290	DOWN
ENSMUSG00000027284	Cdan1	16.8557	26.8790	-0.6732	0.0408	DOWN
ENSMUSG00000028920	Fbxo42	34.5821	55.0887	-0.6717	0.0223	DOWN
ENSMUSG00000042423	Fbrs	34.2070	54.2395	-0.6651	0.0277	DOWN
ENSMUSG00000036634	Mag	39.2830	61.4969	-0.6466	0.0179	DOWN
ENSMUSG00000040430	Pitpnc1	34.3888	53.7607	-0.6446	0.0202	DOWN
ENSMUSG00000022186	Oxct1	50.8914	79.2132	-0.6383	0.0269	DOWN
ENSMUSG00000054051	Ercc6	37.4015	58.0213	-0.6335	0.0377	DOWN
ENSMUSG00000042506	Usp22	59.7421	92.5107	-0.6309	0.0188	DOWN
ENSMUSG00000053093	Myh7	25.8420	39.6610	-0.6180	0.0382	DOWN
ENSMUSG00000042105	Inpp5f	19.1332	29.2772	-0.6137	0.0399	DOWN
ENSMUSG00000062380	Tubb3	105.6667	160.1683	-0.6001	0.0454	DOWN
ENSMUSG00000052609	Plekhg3	50.2937	74.9520	-0.5756	0.0388	DOWN
ENSMUSG00000030795	Fus	11.7129	17.4025	-0.5712	0.0419	DOWN
ENSMUSG00000049577	Zfpm1	91.9451	135.6325	-0.5609	0.0197	DOWN
ENSMUSG00000001440	Kpnb1	41.6718	61.3720	-0.5585	0.0469	DOWN
ENSMUSG00000020882	Cacnb1	132.2108	193.7879	-0.5516	0.0165	DOWN
ENSMUSG00000020936	Nmt1	76.1770	110.0665	-0.5309	0.0331	DOWN
ENSMUSG00000047388	Atmin	58.0898	83.5455	-0.5243	0.0486	DOWN
ENSMUSG00000025034	Trim8	462.8197	618.2397	-0.4177	0.0470	DOWN

Supplementary Table 2
List of the differentially expressed lncRNAs.

lncRNA id	CIPC	Sham	log2FC	Pvalue	up-down
NONMMUT084708.1	1.8125	0.0000	Inf	0.0000	UP
NONMMUT053114.2	1.2187	0.0000	Inf	0.0001	UP
NONMMUT054261.2	1.2738	0.0000	Inf	0.0001	UP
ENSMUST00000145803	1.2320	0.0000	Inf	0.0002	UP
NONMMUT033609.2	1.7701	0.0000	Inf	0.0003	UP
NONMMUT104274.1	1.2441	0.0000	Inf	0.0005	UP
NONMMUT054409.2	0.4321	0.0000	Inf	0.0006	UP
NONMMUT085833.1	0.5775	0.0000	Inf	0.0006	UP
ENSMUST00000180839	0.6041	0.0000	Inf	0.0007	UP
ENSMUST00000130256	0.2715	0.0000	Inf	0.0007	UP
ENSMUST00000181496	0.9163	0.0000	Inf	0.0008	UP
NONMMUT045373.2	0.7975	0.0000	Inf	0.0012	UP
NONMMUT017775.2	0.9807	0.0000	Inf	0.0013	UP
NONMMUT033596.2	0.9723	0.0000	Inf	0.0014	UP
NONMMUT010524.2	0.9040	0.0000	Inf	0.0015	UP
NONMMUT044110.2	1.6275	0.0000	Inf	0.0017	UP
ENSMUST00000145533	1.4414	0.0000	Inf	0.0021	UP
NONMMUT057423.2	0.6179	0.0000	Inf	0.0022	UP
NONMMUT027222.2	0.1987	0.0000	Inf	0.0039	UP
ENSMUST00000193520	0.7392	0.0000	Inf	0.0039	UP
NONMMUT058911.2	0.1874	0.0000	Inf	0.0040	UP
ENSMUST00000181811	0.3477	0.0000	Inf	0.0041	UP
NONMMUT108213.1	0.5714	0.0000	Inf	0.0045	UP
NONMMUT055520.2	0.2119	0.0000	Inf	0.0046	UP
NONMMUT120516.1	0.2976	0.0000	Inf	0.0049	UP
NONMMUT054892.2	0.2627	0.0000	Inf	0.0051	UP
ENSMUST00000188519	0.5946	0.0000	Inf	0.0052	UP
NONMMUT034225.2	0.2682	0.0000	Inf	0.0053	UP
NONMMUT024071.2	0.7633	0.0000	Inf	0.0061	UP
ENSMUST00000181605	0.7331	0.0000	Inf	0.0061	UP
NONMMUT009485.2	1.5533	0.0000	Inf	0.0063	UP
ENSMUST00000200761	0.2065	0.0000	Inf	0.0064	UP
NONMMUT008057.2	0.5034	0.0000	Inf	0.0072	UP
NONMMUT049808.2	0.9333	0.0000	Inf	0.0077	UP
NONMMUT132397.1	0.7389	0.0000	Inf	0.0078	UP
NONMMUT124741.1	0.4533	0.0000	Inf	0.0084	UP
NONMMUT076551.1	0.4048	0.0000	Inf	0.0085	UP
NONMMUT033961.2	0.7564	0.0000	Inf	0.0097	UP
NONMMUT125003.1	0.5475	0.0000	Inf	0.0098	UP
NONMMUT100081.1	0.3210	0.0000	Inf	0.0109	UP
NONMMUT055156.2	0.3113	0.0000	Inf	0.0112	UP
NONMMUT013383.2	0.2742	0.0000	Inf	0.0113	UP
NONMMUT068100.2	0.1327	0.0000	Inf	0.0115	UP
NONMMUT055118.2	0.7619	0.0000	Inf	0.0118	UP
NONMMUT064545.2	0.6730	0.0000	Inf	0.0120	UP
NONMMUT122370.1	0.2339	0.0000	Inf	0.0121	UP

NONMMUT029512.2	0.3168	0.0000	Inf	0.0122	UP
NONMMUT028740.2	0.6121	0.0000	Inf	0.0123	UP
ENSMUST00000174287	0.4962	0.0000	Inf	0.0136	UP
NONMMUT125636.1	0.1840	0.0000	Inf	0.0138	UP
NONMMUT107755.1	0.1144	0.0000	Inf	0.0139	UP
NONMMUT048018.2	2.3073	0.0000	Inf	0.0145	UP
NONMMUT028257.2	0.1736	0.0000	Inf	0.0154	UP
ENSMUST00000188976	0.4390	0.0000	Inf	0.0154	UP
NONMMUT128131.1	0.1414	0.0000	Inf	0.0157	UP
NONMMUT102509.1	0.8913	0.0000	Inf	0.0159	UP
NONMMUT048434.2	0.8941	0.0000	Inf	0.0160	UP
NONMMUT050648.2	0.2754	0.0000	Inf	0.0165	UP
NONMMUT062483.2	0.3733	0.0000	Inf	0.0169	UP
NONMMUT118190.1	0.2385	0.0000	Inf	0.0172	UP
NONMMUT075979.1	0.4119	0.0000	Inf	0.0174	UP
NONMMUT117330.1	0.3996	0.0000	Inf	0.0174	UP
NONMMUT073304.2	1.9082	0.0000	Inf	0.0178	UP
NONMMUT067117.2	0.1206	0.0000	Inf	0.0180	UP
NONMMUT050992.2	0.3339	0.0000	Inf	0.0183	UP
NONMMUT013306.2	0.2455	0.0000	Inf	0.0186	UP
NONMMUT016019.2	0.1425	0.0000	Inf	0.0189	UP
MSTRG.135855.1	1.4291	0.0000	Inf	0.0191	UP
NONMMUT099995.1	0.1797	0.0000	Inf	0.0192	UP
NONMMUT108545.1	0.3107	0.0000	Inf	0.0194	UP
NONMMUT061822.2	0.9428	0.0000	Inf	0.0194	UP
NONMMUT069401.2	0.1670	0.0000	Inf	0.0195	UP
NONMMUT106149.1	0.3637	0.0000	Inf	0.0196	UP
NONMMUT024436.2	0.3219	0.0000	Inf	0.0198	UP
NONMMUT097421.1	0.5554	0.0000	Inf	0.0198	UP
NONMMUT000263.2	0.1962	0.0000	Inf	0.0202	UP
ENSMUST00000161043	0.4695	0.0000	Inf	0.0202	UP
NONMMUT062294.2	0.2061	0.0000	Inf	0.0206	UP
NONMMUT090261.1	0.1800	0.0000	Inf	0.0209	UP
NONMMUT098098.1	0.7854	0.0000	Inf	0.0215	UP
NONMMUT057117.2	0.3881	0.0000	Inf	0.0216	UP
NONMMUT131922.1	0.0815	0.0000	Inf	0.0219	UP
MSTRG.99438.1	2.0064	0.0000	Inf	0.0223	UP
NONMMUT102270.1	0.2860	0.0000	Inf	0.0224	UP
NONMMUT065947.2	0.6062	0.0000	Inf	0.0225	UP
NONMMUT009212.2	0.1420	0.0000	Inf	0.0229	UP
NONMMUT051645.2	1.0758	0.0000	Inf	0.0234	UP
NONMMUT069564.2	0.4055	0.0000	Inf	0.0237	UP
NONMMUT131745.1	0.1605	0.0000	Inf	0.0257	UP
NONMMUT075736.1	0.3571	0.0000	Inf	0.0261	UP
NONMMUT125201.1	0.4952	0.0000	Inf	0.0264	UP
NONMMUT002687.2	0.2900	0.0000	Inf	0.0269	UP
NONMMUT122522.1	0.1828	0.0000	Inf	0.0270	UP
ENSMUST00000150604	0.4477	0.0000	Inf	0.0271	UP
NONMMUT133900.1	0.3946	0.0000	Inf	0.0273	UP

ENSMUST00000156926	0.6000	0.0000	Inf	0.0283	UP
NONMMUT032482.2	0.8470	0.0000	Inf	0.0284	UP
NONMMUT053707.2	0.1806	0.0000	Inf	0.0286	UP
NONMMUT129448.1	0.8790	0.0000	Inf	0.0289	UP
NONMMUT003550.2	0.3203	0.0000	Inf	0.0297	UP
NONMMUT103053.1	0.6243	0.0000	Inf	0.0301	UP
NONMMUT082368.1	0.8825	0.0000	Inf	0.0303	UP
NONMMUT135616.1	0.1216	0.0000	Inf	0.0309	UP
NONMMUT032395.2	0.1719	0.0000	Inf	0.0310	UP
NONMMUT034784.2	0.2155	0.0000	Inf	0.0311	UP
ENSMUST00000183670	1.1389	0.0000	Inf	0.0314	UP
NONMMUT075271.1	0.3526	0.0000	Inf	0.0319	UP
NONMMUT122981.1	0.8196	0.0000	Inf	0.0322	UP
NONMMUT112559.1	0.4366	0.0000	Inf	0.0322	UP
NONMMUT037672.2	1.6631	0.0000	Inf	0.0326	UP
NONMMUT029641.2	0.4452	0.0000	Inf	0.0327	UP
NONMMUT130852.1	0.4704	0.0000	Inf	0.0327	UP
NONMMUT074145.2	0.1828	0.0000	Inf	0.0332	UP
NONMMUT086008.1	0.5598	0.0000	Inf	0.0333	UP
ENSMUST00000195414	0.5456	0.0000	Inf	0.0333	UP
NONMMUT078552.1	0.2634	0.0000	Inf	0.0334	UP
NONMMUT049703.2	0.4165	0.0000	Inf	0.0338	UP
NONMMUT129408.1	0.4600	0.0000	Inf	0.0338	UP
NONMMUT078565.1	0.3057	0.0000	Inf	0.0341	UP
ENSMUST00000129007	0.3079	0.0000	Inf	0.0343	UP
NONMMUT027208.2	0.1719	0.0000	Inf	0.0344	UP
NONMMUT107912.1	0.1201	0.0000	Inf	0.0345	UP
NONMMUT032283.2	0.0986	0.0000	Inf	0.0345	UP
NONMMUT078176.1	0.2741	0.0000	Inf	0.0349	UP
NONMMUT102380.1	0.4880	0.0000	Inf	0.0353	UP
NONMMUT095849.1	0.4080	0.0000	Inf	0.0358	UP
NONMMUT129133.1	0.1955	0.0000	Inf	0.0360	UP
NONMMUT011770.2	0.3970	0.0000	Inf	0.0362	UP
NONMMUT094869.1	0.1334	0.0000	Inf	0.0365	UP
NONMMUT133118.1	0.2524	0.0000	Inf	0.0375	UP
NONMMUT012986.2	0.2755	0.0000	Inf	0.0376	UP
NONMMUT092345.1	0.5425	0.0000	Inf	0.0379	UP
NONMMUT075366.1	0.1269	0.0000	Inf	0.0381	UP
NONMMUT066534.2	0.1393	0.0000	Inf	0.0381	UP
NONMMUT041171.2	0.4380	0.0000	Inf	0.0385	UP
NONMMUT000053.2	0.3108	0.0000	Inf	0.0385	UP
NONMMUT028683.2	0.1750	0.0000	Inf	0.0387	UP
NONMMUT091505.1	0.2075	0.0000	Inf	0.0393	UP
NONMMUT076175.1	0.4364	0.0000	Inf	0.0398	UP
ENSMUST00000180477	0.7238	0.0000	Inf	0.0398	UP
ENSMUST00000154354	0.5477	0.0000	Inf	0.0399	UP
NONMMUT074537.2	0.3461	0.0000	Inf	0.0406	UP
NONMMUT113106.1	0.1793	0.0000	Inf	0.0412	UP
NONMMUT106093.1	0.1634	0.0000	Inf	0.0414	UP

NONMMUT104193.1	0.3847	0.0000	Inf	0.0415	UP
NONMMUT113070.1	0.3784	0.0000	Inf	0.0425	UP
NONMMUT093974.1	0.3159	0.0000	Inf	0.0426	UP
MSTRG.98309.1	1.3587	0.0000	Inf	0.0426	UP
NONMMUT023049.2	0.2423	0.0000	Inf	0.0426	UP
NONMMUT027862.2	0.1558	0.0000	Inf	0.0426	UP
NONMMUT117578.1	0.3690	0.0000	Inf	0.0427	UP
ENSMUST00000153785	0.2886	0.0000	Inf	0.0432	UP
NONMMUT107418.1	0.2768	0.0000	Inf	0.0436	UP
ENSMUST00000147420	0.1823	0.0000	Inf	0.0441	UP
NONMMUT093014.1	0.3940	0.0000	Inf	0.0441	UP
NONMMUT109950.1	0.1302	0.0000	Inf	0.0445	UP
NONMMUT058913.2	0.0368	0.0000	Inf	0.0451	UP
NONMMUT090381.1	0.1768	0.0000	Inf	0.0454	UP
NONMMUT053900.2	0.5400	0.0000	Inf	0.0456	UP
NONMMUT086157.1	0.2239	0.0000	Inf	0.0457	UP
NONMMUT055060.2	0.2830	0.0000	Inf	0.0458	UP
NONMMUT018146.2	0.1858	0.0000	Inf	0.0463	UP
NONMMUT025734.2	0.1539	0.0000	Inf	0.0466	UP
NONMMUT125656.1	0.1203	0.0000	Inf	0.0468	UP
NONMMUT085225.1	0.1880	0.0000	Inf	0.0471	UP
NONMMUT131766.1	0.5629	0.0000	Inf	0.0474	UP
NONMMUT003638.2	0.1005	0.0000	Inf	0.0477	UP
NONMMUT130984.1	0.4929	0.0000	Inf	0.0482	UP
NONMMUT076902.1	0.2257	0.0000	Inf	0.0484	UP
NONMMUT045676.2	0.2114	0.0000	Inf	0.0484	UP
NONMMUT117721.1	0.6402	0.0000	Inf	0.0485	UP
NONMMUT103106.1	0.6358	0.0000	Inf	0.0487	UP
NONMMUT127092.1	0.3889	0.0000	Inf	0.0487	UP
NONMMUT137171.1	0.0707	0.0000	Inf	0.0488	UP
NONMMUT058299.2	1.4636	0.0000	Inf	0.0490	UP
NONMMUT051017.2	0.5706	0.0000	Inf	0.0492	UP
NONMMUT059676.2	0.5536	0.0000	Inf	0.0492	UP
NONMMUT054298.2	1.3296	0.0000	Inf	0.0492	UP
NONMMUT037010.2	0.1800	0.0000	Inf	0.0493	UP
NONMMUT100054.1	0.3236	0.0000	Inf	0.0498	UP
NONMMUT051354.2	0.3696	0.0000	Inf	0.0499	UP
NONMMUT109796.1	0.8135	0.0060	7.0763	0.0005	UP
ENSMUST00000181535	1.8518	0.0151	6.9408	0.0107	UP
NONMMUT041264.2	1.2305	0.0126	6.6047	0.0158	UP
NONMMUT084500.1	0.5092	0.0081	5.9821	0.0035	UP
NONMMUT031944.2	0.1963	0.0032	5.9315	0.0282	UP
NONMMUT084920.1	0.2365	0.0041	5.8667	0.0031	UP
NONMMUT082657.1	1.2241	0.0219	5.8059	0.0036	UP
NONMMUT113501.1	1.2443	0.0226	5.7830	0.0047	UP
NONMMUT081453.1	1.1121	0.0204	5.7697	0.0163	UP
ENSMUST00000146644	0.2609	0.0051	5.6839	0.0405	UP
NONMMUT066321.2	0.3556	0.0072	5.6180	0.0471	UP
NONMMUT078689.1	1.1116	0.0229	5.5992	0.0016	UP

NONMMUT122915.1	0.1505	0.0033	5.5145	0.0051	UP
NONMMUT092914.1	0.2786	0.0061	5.5048	0.0152	UP
NONMMUT056389.2	0.2868	0.0063	5.5040	0.0331	UP
NONMMUT022169.2	0.2572	0.0059	5.4572	0.0440	UP
MSTRG.111437.2	1.6794	0.0391	5.4237	0.0379	UP
MSTRG.47437.2	13.5181	0.3209	5.3968	0.0031	UP
NONMMUT086127.1	0.7322	0.0183	5.3226	0.0001	UP
NONMMUT069230.2	0.2681	0.0067	5.3197	0.0098	UP
NONMMUT032580.2	1.4344	0.0362	5.3099	0.0028	UP
NONMMUT059498.2	0.2358	0.0061	5.2695	0.0217	UP
NONMMUT021303.2	0.2100	0.0055	5.2615	0.0450	UP
ENSMUST00000132090	0.2418	0.0065	5.2061	0.0166	UP
NONMMUT029757.2	0.2350	0.0065	5.1718	0.0268	UP
NONMMUT093146.1	0.4579	0.0134	5.0987	0.0325	UP
ENSMUST00000194481	0.4092	0.0122	5.0714	0.0329	UP
NONMMUT022826.2	0.3805	0.0115	5.0520	0.0160	UP
NONMMUT023026.2	0.5881	0.0180	5.0282	0.0354	UP
NONMMUT094879.1	0.2721	0.0085	5.0013	0.0006	UP
NONMMUT060147.2	0.5887	0.0189	4.9573	0.0276	UP
NONMMUT038154.2	0.1193	0.0039	4.9456	0.0428	UP
MSTRG.148250.1	0.8801	0.0286	4.9450	0.0283	UP
NONMMUT045393.2	0.5207	0.0173	4.9150	0.0338	UP
NONMMUT098173.1	0.2353	0.0078	4.9111	0.0097	UP
NONMMUT084511.1	0.8786	0.0294	4.9034	0.0087	UP
NONMMUT028752.2	0.3832	0.0129	4.8960	0.0156	UP
NONMMUT069296.2	0.3402	0.0120	4.8283	0.0273	UP
NONMMUT131991.1	1.5730	0.0559	4.8154	0.0371	UP
ENSMUST00000180955	0.1247	0.0045	4.8088	0.0364	UP
NONMMUT115685.1	0.1288	0.0046	4.8056	0.0373	UP
NONMMUT104386.1	0.1080	0.0043	4.6667	0.0033	UP
NONMMUT034417.2	0.4201	0.0167	4.6542	0.0042	UP
NONMMUT125875.1	0.3193	0.0127	4.6497	0.0413	UP
NONMMUT035095.2	0.3400	0.0137	4.6348	0.0312	UP
NONMMUT024062.2	0.7317	0.0298	4.6191	0.0397	UP
NONMMUT053411.2	0.2922	0.0119	4.6147	0.0052	UP
NONMMUT028627.2	0.2910	0.0119	4.6070	0.0388	UP
NONMMUT067203.2	0.8334	0.0347	4.5876	0.0312	UP
NONMMUT017381.2	0.3435	0.0143	4.5843	0.0243	UP
NONMMUT130662.1	0.3084	0.0135	4.5144	0.0170	UP
NONMMUT135571.1	0.4517	0.0201	4.4931	0.0075	UP
NONMMUT008178.2	1.1067	0.0503	4.4582	0.0100	UP
NONMMUT109112.1	0.2948	0.0139	4.4022	0.0266	UP
NONMMUT050016.2	0.1436	0.0069	4.3884	0.0224	UP
NONMMUT093933.1	0.4132	0.0201	4.3638	0.0164	UP
NONMMUT019629.2	5.6653	0.2762	4.3586	0.0034	UP
NONMMUT012991.2	0.5842	0.0299	4.2889	0.0424	UP
ENSMUST00000133270	1.2765	0.0660	4.2740	0.0151	UP
ENSMUST00000113671	0.8266	0.0436	4.2449	0.0243	UP
NONMMUT034485.2	1.3417	0.0721	4.2180	0.0283	UP

NONMMUT008216.2	1.0837	0.0613	4.1445	0.0093	UP
NONMMUT029636.2	15.8179	0.8956	4.1426	0.0212	UP
ENSMUST00000203443	0.5803	0.0334	4.1172	0.0477	UP
NONMMUT001381.2	0.2206	0.0128	4.1052	0.0371	UP
NONMMUT065401.2	0.3094	0.0183	4.0817	0.0361	UP
NONMMUT046803.2	0.3882	0.0233	4.0571	0.0019	UP
NONMMUT013073.2	0.6495	0.0406	3.9992	0.0032	UP
NONMMUT032173.2	0.5236	0.0328	3.9965	0.0070	UP
NONMMUT082670.1	0.4609	0.0289	3.9948	0.0001	UP
NONMMUT061228.2	0.8633	0.0546	3.9815	0.0141	UP
NONMMUT035084.2	0.7233	0.0463	3.9655	0.0249	UP
NONMMUT073412.2	0.2130	0.0137	3.9542	0.0361	UP
NONMMUT016327.2	0.5602	0.0363	3.9482	0.0077	UP
NONMMUT135545.1	0.3465	0.0228	3.9278	0.0403	UP
NONMMUT025771.2	0.2417	0.0161	3.9043	0.0063	UP
NONMMUT094701.1	0.6578	0.0440	3.9034	0.0490	UP
NONMMUT119131.1	0.4974	0.0336	3.8899	0.0232	UP
MSTRG.52935.1	2.5853	0.1826	3.8234	0.0038	UP
MSTRG.91401.1	2.8301	0.2075	3.7697	0.0384	UP
NONMMUT039484.2	3.3524	0.2492	3.7499	0.0096	UP
NONMMUT069865.2	0.2356	0.0175	3.7481	0.0320	UP
ENSMUST00000163493	1.0958	0.0821	3.7382	0.0017	UP
NONMMUT068261.2	1.6260	0.1232	3.7224	0.0188	UP
NONMMUT050236.2	0.5147	0.0394	3.7086	0.0164	UP
NONMMUT095265.1	0.4698	0.0360	3.7063	0.0355	UP
NONMMUT070191.2	0.1413	0.0110	3.6843	0.0209	UP
NONMMUT051065.2	1.3018	0.1018	3.6765	0.0080	UP
NONMMUT064883.2	0.4712	0.0370	3.6725	0.0354	UP
NONMMUT126672.1	1.5186	0.1212	3.6476	0.0128	UP
NONMMUT022997.2	0.7063	0.0566	3.6404	0.0008	UP
NONMMUT031182.2	0.6170	0.0501	3.6232	0.0348	UP
NONMMUT068130.2	2.1199	0.1722	3.6217	0.0351	UP
NONMMUT088753.1	0.2420	0.0199	3.6033	0.0284	UP
NONMMUT059003.2	0.1115	0.0094	3.5746	0.0463	UP
NONMMUT084131.1	0.6363	0.0539	3.5613	0.0452	UP
NONMMUT011867.2	0.3773	0.0330	3.5132	0.0301	UP
NONMMUT137163.1	0.3482	0.0310	3.4894	0.0470	UP
NONMMUT026441.2	6.9713	0.6290	3.4703	0.0024	UP
NONMMUT086696.1	0.8802	0.0809	3.4440	0.0372	UP
NONMMUT005414.2	1.0979	0.1023	3.4244	0.0013	UP
NONMMUT094100.1	0.6177	0.0578	3.4173	0.0470	UP
NONMMUT127068.1	0.7047	0.0666	3.4036	0.0152	UP
NONMMUT054536.2	0.5241	0.0500	3.3895	0.0161	UP
NONMMUT018741.2	1.6770	0.1629	3.3635	0.0021	UP
NONMMUT086137.1	0.3578	0.0356	3.3309	0.0106	UP
NONMMUT003696.2	0.6150	0.0624	3.3003	0.0044	UP
NONMMUT008470.2	3.3134	0.3404	3.2828	0.0028	UP
NONMMUT019759.2	0.4189	0.0432	3.2763	0.0248	UP
NONMMUT020044.2	0.2131	0.0221	3.2707	0.0479	UP

NONMMUT089698.1	0.2434	0.0255	3.2544	0.0289	UP
NONMMUT012557.2	0.2276	0.0244	3.2231	0.0253	UP
NONMMUT068696.2	2.8728	0.3126	3.1999	0.0099	UP
NONMMUT075061.1	0.7072	0.0786	3.1700	0.0117	UP
NONMMUT047800.2	0.9141	0.1043	3.1316	0.0276	UP
NONMMUT119134.1	0.3617	0.0419	3.1108	0.0332	UP
ENSMUST00000197836	0.1887	0.0232	3.0243	0.0468	UP
NONMMUT024336.2	5.4204	0.6728	3.0101	0.0208	UP
ENSMUST00000148066	0.4813	0.0599	3.0061	0.0292	UP
NONMMUT057231.2	0.5859	0.0774	2.9197	0.0163	UP
NONMMUT006764.2	0.3749	0.0499	2.9107	0.0159	UP
NONMMUT026329.2	0.4120	0.0554	2.8945	0.0330	UP
MSTRG.44458.2	5.1795	0.6973	2.8929	0.0034	UP
NONMMUT056398.2	0.6239	0.0852	2.8719	0.0126	UP
NONMMUT035147.2	0.2143	0.0304	2.8154	0.0485	UP
NONMMUT120489.1	0.2977	0.0427	2.8002	0.0412	UP
NONMMUT024256.2	1.4172	0.2066	2.7783	0.0158	UP
NONMMUT014207.2	0.3756	0.0554	2.7616	0.0343	UP
NONMMUT056810.2	0.3752	0.0580	2.6935	0.0373	UP
NONMMUT064705.2	1.5038	0.2328	2.6918	0.0115	UP
NONMMUT018829.2	0.1460	0.0229	2.6715	0.0438	UP
NONMMUT069037.2	0.4457	0.0702	2.6670	0.0427	UP
NONMMUT099508.1	2.3030	0.3673	2.6485	0.0275	UP
ENSMUST00000185564	0.5123	0.0837	2.6141	0.0155	UP
NONMMUT062889.2	1.9340	0.3189	2.6004	0.0109	UP
NONMMUT064558.2	0.2097	0.0347	2.5964	0.0209	UP
NONMMUT060075.2	13.1308	2.1873	2.5857	0.0113	UP
NONMMUT068884.2	0.8628	0.1488	2.5352	0.0417	UP
NONMMUT026272.2	0.8099	0.1436	2.4956	0.0360	UP
NONMMUT033949.2	0.4861	0.0867	2.4875	0.0268	UP
NONMMUT098863.1	2.4879	0.4489	2.4706	0.0039	UP
NONMMUT063472.2	0.2525	0.0457	2.4664	0.0475	UP
MSTRG.157605.1	1.8453	0.3474	2.4091	0.0067	UP
NONMMUT066649.2	0.4247	0.0809	2.3927	0.0271	UP
NONMMUT007253.2	0.4479	0.0864	2.3745	0.0354	UP
NONMMUT008056.2	1.6504	0.3190	2.3711	0.0190	UP
NONMMUT002077.2	2.6928	0.5257	2.3568	0.0432	UP
NONMMUT106634.1	0.4584	0.0902	2.3454	0.0242	UP
NONMMUT031987.2	0.5601	0.1107	2.3385	0.0124	UP
NONMMUT013242.2	0.6403	0.1287	2.3144	0.0216	UP
NONMMUT005748.2	0.4440	0.0903	2.2984	0.0414	UP
NONMMUT048560.2	1.2891	0.2630	2.2932	0.0121	UP
NONMMUT068747.2	2.4900	0.5150	2.2735	0.0036	UP
NONMMUT001985.2	0.3210	0.0667	2.2669	0.0455	UP
NONMMUT069498.2	0.6063	0.1265	2.2610	0.0454	UP
NONMMUT066075.2	0.7183	0.1515	2.2455	0.0347	UP
NONMMUT033521.2	1.4147	0.3010	2.2327	0.0126	UP
ENSMUST00000194141	0.4303	0.0917	2.2302	0.0460	UP
NONMMUT050504.2	0.3890	0.0837	2.2167	0.0251	UP

NONMMUT069825.2	0.3793	0.0830	2.1926	0.0337	UP
NONMMUT072366.2	0.5651	0.1239	2.1894	0.0297	UP
NONMMUT050421.2	1.0619	0.2367	2.1653	0.0223	UP
NONMMUT050980.2	0.6304	0.1449	2.1207	0.0338	UP
NONMMUT062468.2	0.6413	0.1526	2.0711	0.0317	UP
NONMMUT005000.2	0.4248	0.1038	2.0334	0.0462	UP
ENSMUST00000192285	0.8863	0.2233	1.9887	0.0199	UP
NONMMUT057744.2	4.9778	1.2719	1.9686	0.0251	UP
NONMMUT068410.2	0.5150	0.1324	1.9599	0.0362	UP
NONMMUT011172.2	2.7786	0.7244	1.9396	0.0112	UP
NONMMUT037634.2	1.6942	0.4426	1.9364	0.0267	UP
NONMMUT032200.2	0.7143	0.1869	1.9344	0.0254	UP
NONMMUT008006.2	2.5252	0.6681	1.9182	0.0253	UP
NONMMUT025233.2	0.6462	0.1722	1.9081	0.0282	UP
NONMMUT015708.2	2.8527	0.7654	1.8980	0.0333	UP
MSTRG.15989.1	3.2198	0.8669	1.8930	0.0413	UP
NONMMUT039924.2	0.9497	0.2603	1.8676	0.0068	UP
NONMMUT057646.2	1.2683	0.3551	1.8368	0.0415	UP
NONMMUT026614.2	0.9848	0.2797	1.8158	0.0245	UP
ENSMUST00000194151	0.5394	0.1566	1.7843	0.0245	UP
NONMMUT064355.2	2.2360	0.6576	1.7656	0.0194	UP
NONMMUT117260.1	0.5607	0.1710	1.7130	0.0208	UP
NONMMUT007982.2	0.7673	0.2370	1.6951	0.0483	UP
ENSMUST00000191153	2.0183	0.6246	1.6923	0.0306	UP
NONMMUT015629.2	1.2286	0.3852	1.6732	0.0494	UP
NONMMUT135729.1	0.0914	0.0291	1.6519	0.0498	UP
NONMMUT012202.2	0.3731	0.1225	1.6071	0.0484	UP
NONMMUT025361.2	0.9406	0.3285	1.5178	0.0339	UP
NONMMUT070862.2	1.1283	0.4024	1.4876	0.0318	UP
NONMMUT022479.2	1.6117	0.5826	1.4679	0.0496	UP
NONMMUT004140.2	0.8028	0.2910	1.4641	0.0487	UP
NONMMUT033869.2	10.0891	3.7184	1.4400	0.0147	UP
NONMMUT035195.2	2.8026	1.0858	1.3680	0.0477	UP
NONMMUT066069.2	0.6914	0.2877	1.2649	0.0471	UP
ENSMUST00000206829	0.0000	0.8434	-Inf	0.0000	DOWN
NONMMUT047694.2	0.0000	1.0572	-Inf	0.0000	DOWN
NONMMUT053709.2	0.0000	0.3655	-Inf	0.0001	DOWN
NONMMUT033610.2	0.0000	26.5307	-Inf	0.0001	DOWN
NONMMUT073196.2	0.0000	0.3969	-Inf	0.0001	DOWN
NONMMUT061671.2	0.0000	0.2186	-Inf	0.0001	DOWN
NONMMUT066153.2	0.0000	1.0413	-Inf	0.0003	DOWN
NONMMUT022911.2	0.0000	7.5404	-Inf	0.0003	DOWN
NONMMUT060524.2	0.0000	2.3502	-Inf	0.0003	DOWN
NONMMUT008307.2	0.0000	0.5228	-Inf	0.0005	DOWN
NONMMUT133514.1	0.0000	0.7117	-Inf	0.0006	DOWN
NONMMUT073112.2	0.0000	6.2571	-Inf	0.0007	DOWN
NONMMUT011164.2	0.0000	0.3803	-Inf	0.0009	DOWN
NONMMUT032843.2	0.0000	1.3250	-Inf	0.0010	DOWN
ENSMUST00000128904	0.0000	1.3540	-Inf	0.0011	DOWN

ENSMUST00000139612	0.0000	1.6315	-Inf	0.0013	DOWN
NONMMUT041715.2	0.0000	0.3486	-Inf	0.0013	DOWN
NONMMUT108382.1	0.0000	1.1500	-Inf	0.0015	DOWN
NONMMUT033611.2	0.0000	4.3790	-Inf	0.0015	DOWN
NONMMUT063702.2	0.0000	0.4632	-Inf	0.0015	DOWN
NONMMUT056082.2	0.0000	0.5416	-Inf	0.0015	DOWN
NONMMUT012897.2	0.0000	0.3570	-Inf	0.0020	DOWN
ENSMUST00000202292	0.0000	0.2105	-Inf	0.0021	DOWN
NONMMUT022740.2	0.0000	0.2732	-Inf	0.0022	DOWN
NONMMUT078852.1	0.0000	0.7927	-Inf	0.0023	DOWN
NONMMUT044728.2	0.0000	0.2093	-Inf	0.0023	DOWN
NONMMUT068833.2	0.0000	0.6910	-Inf	0.0044	DOWN
NONMMUT041938.2	0.0000	0.5903	-Inf	0.0045	DOWN
NONMMUT030467.2	0.0000	0.6610	-Inf	0.0048	DOWN
ENSMUST00000187361	0.0000	0.8859	-Inf	0.0053	DOWN
NONMMUT013885.2	0.0000	0.3018	-Inf	0.0057	DOWN
NONMMUT097914.1	0.0000	0.3033	-Inf	0.0059	DOWN
NONMMUT051689.2	0.0000	1.1140	-Inf	0.0062	DOWN
NONMMUT132020.1	0.0000	0.0706	-Inf	0.0063	DOWN
NONMMUT053259.2	0.0000	0.0872	-Inf	0.0069	DOWN
NONMMUT048791.2	0.0000	0.4968	-Inf	0.0071	DOWN
NONMMUT132955.1	0.0000	1.0190	-Inf	0.0074	DOWN
NONMMUT118365.1	0.0000	0.5388	-Inf	0.0078	DOWN
NONMMUT073114.2	0.0000	1.8726	-Inf	0.0079	DOWN
NONMMUT039241.2	0.0000	0.6175	-Inf	0.0084	DOWN
NONMMUT047043.2	0.0000	0.6680	-Inf	0.0085	DOWN
NONMMUT018143.2	0.0000	0.4016	-Inf	0.0086	DOWN
NONMMUT005608.2	0.0000	0.2738	-Inf	0.0092	DOWN
NONMMUT070470.2	0.0000	0.2877	-Inf	0.0093	DOWN
NONMMUT009035.2	0.0000	0.2600	-Inf	0.0096	DOWN
NONMMUT133517.1	0.0000	2.1278	-Inf	0.0103	DOWN
NONMMUT120209.1	0.0000	0.4552	-Inf	0.0104	DOWN
NONMMUT050432.2	0.0000	0.3199	-Inf	0.0105	DOWN
NONMMUT053655.2	0.0000	0.3957	-Inf	0.0118	DOWN
NONMMUT018000.2	0.0000	0.4483	-Inf	0.0119	DOWN
NONMMUT014734.2	0.0000	0.7166	-Inf	0.0121	DOWN
NONMMUT052110.2	0.0000	1.9243	-Inf	0.0123	DOWN
NONMMUT031454.2	0.0000	0.9896	-Inf	0.0125	DOWN
ENSMUST00000180833	0.0000	1.5491	-Inf	0.0126	DOWN
NONMMUT055252.2	0.0000	1.1367	-Inf	0.0126	DOWN
NONMMUT122436.1	0.0000	0.1143	-Inf	0.0130	DOWN
NONMMUT136424.1	0.0000	0.7236	-Inf	0.0131	DOWN
NONMMUT133849.1	0.0000	0.8313	-Inf	0.0132	DOWN
NONMMUT013598.2	0.0000	0.4058	-Inf	0.0134	DOWN
NONMMUT105727.1	0.0000	0.5650	-Inf	0.0144	DOWN
ENSMUST00000197034	0.0000	0.2260	-Inf	0.0152	DOWN
NONMMUT121126.1	0.0000	0.3213	-Inf	0.0159	DOWN
NONMMUT137088.1	0.0000	0.2067	-Inf	0.0160	DOWN
NONMMUT015078.2	0.0000	0.2610	-Inf	0.0164	DOWN

NONMMUT071044.2	0.0000	0.2178	-Inf	0.0173	DOWN
NONMMUT036265.2	0.0000	0.6403	-Inf	0.0176	DOWN
NONMMUT036263.2	0.0000	0.5556	-Inf	0.0176	DOWN
NONMMUT016984.2	0.0000	0.2735	-Inf	0.0180	DOWN
NONMMUT067543.2	0.0000	0.5938	-Inf	0.0184	DOWN
NONMMUT134676.1	0.0000	0.0983	-Inf	0.0187	DOWN
ENSMUST00000167834	0.0000	0.8575	-Inf	0.0189	DOWN
ENSMUST00000196705	0.0000	0.3130	-Inf	0.0197	DOWN
ENSMUST00000204444	0.0000	0.3492	-Inf	0.0204	DOWN
NONMMUT068779.2	0.0000	0.1870	-Inf	0.0207	DOWN
NONMMUT008243.2	0.0000	0.5769	-Inf	0.0219	DOWN
NONMMUT012049.2	0.0000	0.4986	-Inf	0.0224	DOWN
NONMMUT111879.1	0.0000	0.9094	-Inf	0.0225	DOWN
NONMMUT033047.2	0.0000	0.4767	-Inf	0.0226	DOWN
NONMMUT111881.1	0.0000	0.5266	-Inf	0.0228	DOWN
NONMMUT000173.2	0.0000	0.2464	-Inf	0.0231	DOWN
NONMMUT035109.2	0.0000	0.9526	-Inf	0.0232	DOWN
ENSMUST00000192580	0.0000	0.2946	-Inf	0.0232	DOWN
NONMMUT124166.1	0.0000	0.3477	-Inf	0.0235	DOWN
NONMMUT117501.1	0.0000	0.4655	-Inf	0.0241	DOWN
NONMMUT104276.1	0.0000	0.4845	-Inf	0.0243	DOWN
NONMMUT053016.2	0.0000	0.2228	-Inf	0.0244	DOWN
NONMMUT028491.2	0.0000	0.6754	-Inf	0.0244	DOWN
NONMMUT119628.1	0.0000	0.1378	-Inf	0.0245	DOWN
NONMMUT018917.2	0.0000	0.2081	-Inf	0.0246	DOWN
NONMMUT084391.1	0.0000	2.0505	-Inf	0.0252	DOWN
ENSMUST00000148326	0.0000	1.1883	-Inf	0.0252	DOWN
NONMMUT018147.2	0.0000	0.1839	-Inf	0.0257	DOWN
ENSMUST00000180613	0.0000	0.1648	-Inf	0.0258	DOWN
NONMMUT058472.2	0.0000	0.2573	-Inf	0.0261	DOWN
ENSMUST00000192942	0.0000	0.2906	-Inf	0.0267	DOWN
NONMMUT078567.1	0.0000	0.7399	-Inf	0.0274	DOWN
NONMMUT046699.2	0.0000	0.2290	-Inf	0.0276	DOWN
NONMMUT053807.2	0.0000	0.3605	-Inf	0.0279	DOWN
NONMMUT061190.2	0.0000	0.2942	-Inf	0.0283	DOWN
NONMMUT031687.2	0.0000	0.2218	-Inf	0.0284	DOWN
NONMMUT057398.2	0.0000	0.7838	-Inf	0.0288	DOWN
NONMMUT056201.2	0.0000	0.6576	-Inf	0.0289	DOWN
NONMMUT082985.1	0.0000	0.3908	-Inf	0.0290	DOWN
NONMMUT060050.2	0.0000	0.3283	-Inf	0.0291	DOWN
NONMMUT060464.2	0.0000	0.1801	-Inf	0.0292	DOWN
NONMMUT026621.2	0.0000	0.3711	-Inf	0.0300	DOWN
NONMMUT133794.1	0.0000	0.3902	-Inf	0.0302	DOWN
NONMMUT033254.2	0.0000	1.0174	-Inf	0.0305	DOWN
NONMMUT063255.2	0.0000	0.1723	-Inf	0.0306	DOWN
NONMMUT038127.2	0.0000	0.2345	-Inf	0.0308	DOWN
NONMMUT130949.1	0.0000	0.3393	-Inf	0.0314	DOWN
NONMMUT058554.2	0.0000	0.3663	-Inf	0.0316	DOWN
NONMMUT037414.2	0.0000	0.6648	-Inf	0.0320	DOWN

NONMMUT067065.2	0.0000	0.2258	-Inf	0.0323	DOWN
NONMMUT055167.2	0.0000	0.4047	-Inf	0.0325	DOWN
NONMMUT063316.2	0.0000	0.8970	-Inf	0.0329	DOWN
NONMMUT015092.2	0.0000	0.1585	-Inf	0.0330	DOWN
NONMMUT051567.2	0.0000	2.4552	-Inf	0.0331	DOWN
NONMMUT009701.2	0.0000	0.5650	-Inf	0.0332	DOWN
NONMMUT113811.1	0.0000	0.6055	-Inf	0.0334	DOWN
NONMMUT018242.2	0.0000	0.5948	-Inf	0.0334	DOWN
NONMMUT023089.2	0.0000	0.6023	-Inf	0.0337	DOWN
NONMMUT055931.2	0.0000	0.2170	-Inf	0.0342	DOWN
NONMMUT035065.2	0.0000	0.3618	-Inf	0.0343	DOWN
NONMMUT113324.1	0.0000	0.2589	-Inf	0.0346	DOWN
NONMMUT102035.1	0.0000	0.8947	-Inf	0.0347	DOWN
NONMMUT089711.1	0.0000	0.2609	-Inf	0.0347	DOWN
NONMMUT053637.2	0.0000	0.2381	-Inf	0.0350	DOWN
ENSMUST00000181262	0.0000	0.1674	-Inf	0.0353	DOWN
ENSMUST00000181958	0.0000	0.2102	-Inf	0.0361	DOWN
ENSMUST00000147638	0.0000	0.2152	-Inf	0.0363	DOWN
NONMMUT066749.2	0.0000	0.7943	-Inf	0.0363	DOWN
NONMMUT003918.2	0.0000	0.2459	-Inf	0.0368	DOWN
NONMMUT023525.2	0.0000	0.1310	-Inf	0.0373	DOWN
NONMMUT034734.2	0.0000	0.4695	-Inf	0.0388	DOWN
NONMMUT064976.2	0.0000	0.2950	-Inf	0.0388	DOWN
NONMMUT039458.2	0.0000	0.6275	-Inf	0.0388	DOWN
ENSMUST00000159947	0.0000	1.3169	-Inf	0.0394	DOWN
NONMMUT014904.2	0.0000	1.3975	-Inf	0.0395	DOWN
NONMMUT049530.2	0.0000	1.7397	-Inf	0.0398	DOWN
ENSMUST00000151921	0.0000	0.5744	-Inf	0.0408	DOWN
NONMMUT069390.2	0.0000	0.3752	-Inf	0.0410	DOWN
NONMMUT126998.1	0.0000	0.5282	-Inf	0.0412	DOWN
NONMMUT006101.2	0.0000	1.5680	-Inf	0.0415	DOWN
NONMMUT094386.1	0.0000	0.9232	-Inf	0.0416	DOWN
ENSMUST00000181920	0.0000	0.5946	-Inf	0.0421	DOWN
NONMMUT031490.2	0.0000	0.3563	-Inf	0.0421	DOWN
ENSMUST00000191132	0.0000	1.3027	-Inf	0.0426	DOWN
NONMMUT091401.1	0.0000	0.2155	-Inf	0.0427	DOWN
ENSMUST00000181542	0.0000	1.0766	-Inf	0.0429	DOWN
NONMMUT129952.1	0.0000	0.2605	-Inf	0.0429	DOWN
NONMMUT090492.1	0.0000	0.1199	-Inf	0.0432	DOWN
NONMMUT034985.2	0.0000	0.0806	-Inf	0.0432	DOWN
NONMMUT020357.2	0.0000	0.5252	-Inf	0.0443	DOWN
NONMMUT134475.1	0.0000	0.9571	-Inf	0.0454	DOWN
NONMMUT005141.2	0.0000	0.2290	-Inf	0.0455	DOWN
NONMMUT009564.2	0.0000	1.3264	-Inf	0.0460	DOWN
NONMMUT063829.2	0.0000	1.2602	-Inf	0.0460	DOWN
NONMMUT104335.1	0.0000	0.2126	-Inf	0.0466	DOWN
NONMMUT118930.1	0.0000	0.3019	-Inf	0.0471	DOWN
NONMMUT100395.1	0.0000	0.1609	-Inf	0.0473	DOWN
ENSMUST00000140087	0.0000	0.4748	-Inf	0.0474	DOWN

NONMMUT130267.1	0.0000	0.6204	-Inf	0.0480	DOWN
NONMMUT088556.1	0.0000	0.0941	-Inf	0.0481	DOWN
NONMMUT083079.1	0.0000	0.2128	-Inf	0.0481	DOWN
NONMMUT000584.2	0.0000	0.1310	-Inf	0.0481	DOWN
ENSMUST00000105760	0.0000	1.3526	-Inf	0.0487	DOWN
NONMMUT005230.2	0.0000	0.2644	-Inf	0.0492	DOWN
NONMMUT125200.1	0.0000	0.2101	-Inf	0.0496	DOWN
NONMMUT007295.2	2.8415	9.5785	-1.7532	0.0489	DOWN
NONMMUT002607.2	0.5367	2.0035	-1.9004	0.0268	DOWN
NONMMUT098452.1	0.1530	0.5865	-1.9382	0.0490	DOWN
NONMMUT097333.1	0.1814	0.6961	-1.9402	0.0389	DOWN
NONMMUT008498.2	0.5199	2.0885	-2.0061	0.0274	DOWN
NONMMUT061472.2	1.8536	7.7039	-2.0553	0.0462	DOWN
NONMMUT083940.1	0.5904	2.5050	-2.0851	0.0415	DOWN
NONMMUT068882.2	0.3042	1.3090	-2.1055	0.0405	DOWN
NONMMUT009663.2	0.4200	1.8328	-2.1255	0.0409	DOWN
NONMMUT098077.1	0.0668	0.2950	-2.1438	0.0329	DOWN
NONMMUT097870.1	0.1248	0.5524	-2.1456	0.0382	DOWN
ENSMUST00000180868	0.6058	2.7121	-2.1625	0.0437	DOWN
NONMMUT015358.2	0.3135	1.4093	-2.1682	0.0411	DOWN
NONMMUT008156.2	0.7338	3.4581	-2.2365	0.0458	DOWN
NONMMUT010408.2	0.3753	1.7702	-2.2377	0.0312	DOWN
MSTRG.65064.1	0.5016	2.4977	-2.3161	0.0194	DOWN
NONMMUT025265.2	0.4112	2.0726	-2.3336	0.0333	DOWN
NONMMUT062708.2	0.1080	0.5519	-2.3528	0.0396	DOWN
NONMMUT016129.2	0.0652	0.3385	-2.3754	0.0195	DOWN
NONMMUT009993.2	0.0912	0.4732	-2.3756	0.0257	DOWN
NONMMUT127580.1	0.9716	5.1722	-2.4123	0.0411	DOWN
NONMMUT068834.2	0.5358	2.8706	-2.4215	0.0343	DOWN
NONMMUT004640.2	0.1568	0.8441	-2.4282	0.0248	DOWN
MSTRG.150768.1	0.5319	2.8690	-2.4315	0.0488	DOWN
NONMMUT069098.2	0.1044	0.5631	-2.4315	0.0494	DOWN
NONMMUT062467.2	0.2050	1.1111	-2.4384	0.0321	DOWN
NONMMUT041121.2	0.2166	1.1966	-2.4657	0.0266	DOWN
NONMMUT100807.1	0.4462	2.5121	-2.4932	0.0474	DOWN
NONMMUT011336.2	3.6517	20.5682	-2.4938	0.0496	DOWN
NONMMUT024403.2	0.5188	2.9439	-2.5046	0.0228	DOWN
NONMMUT032543.2	0.2062	1.1708	-2.5051	0.0117	DOWN
NONMMUT098886.1	0.0625	0.3579	-2.5169	0.0181	DOWN
NONMMUT100831.1	0.2727	1.5827	-2.5370	0.0242	DOWN
NONMMUT094335.1	0.3942	2.2979	-2.5434	0.0270	DOWN
ENSMUST00000192004	0.3027	1.8022	-2.5737	0.0467	DOWN
NONMMUT073223.2	0.2696	1.6151	-2.5826	0.0408	DOWN
NONMMUT050398.2	0.1286	0.7706	-2.5831	0.0279	DOWN
NONMMUT011321.2	0.1653	1.0128	-2.6149	0.0386	DOWN
ENSMUST00000180410	0.1282	0.7894	-2.6225	0.0254	DOWN
NONMMUT021928.2	0.1757	1.0853	-2.6268	0.0443	DOWN
NONMMUT036036.2	0.1138	0.7125	-2.6461	0.0475	DOWN
ENSMUST00000196037	0.1144	0.7292	-2.6725	0.0344	DOWN

NONMMUT029888.2	0.0553	0.3562	-2.6882	0.0206	DOWN
NONMMUT058162.2	0.0699	0.4512	-2.6903	0.0471	DOWN
NONMMUT017932.2	0.0624	0.4038	-2.6944	0.0314	DOWN
ENSMUST00000202893	0.1218	0.7896	-2.6965	0.0463	DOWN
NONMMUT004928.2	0.0953	0.6219	-2.7066	0.0241	DOWN
NONMMUT064134.2	0.2317	1.5194	-2.7130	0.0334	DOWN
ENSMUST00000193748	0.1703	1.1320	-2.7327	0.0250	DOWN
NONMMUT127848.1	0.0747	0.4969	-2.7340	0.0302	DOWN
NONMMUT124335.1	0.0474	0.3207	-2.7587	0.0200	DOWN
NONMMUT107511.1	0.0950	0.6464	-2.7666	0.0281	DOWN
NONMMUT117648.1	0.0207	0.1434	-2.7934	0.0442	DOWN
NONMMUT026702.2	0.0702	0.4963	-2.8223	0.0214	DOWN
NONMMUT051590.2	0.0479	0.3415	-2.8332	0.0262	DOWN
NONMMUT068303.2	0.0704	0.5216	-2.8893	0.0128	DOWN
NONMMUT043849.2	0.0733	0.5433	-2.8895	0.0490	DOWN
NONMMUT082827.1	0.2133	1.5878	-2.8959	0.0372	DOWN
NONMMUT044427.2	0.0949	0.7071	-2.8968	0.0416	DOWN
NONMMUT007650.2	0.1090	0.8253	-2.9210	0.0086	DOWN
NONMMUT029130.2	0.1428	1.0909	-2.9335	0.0094	DOWN
ENSMUST00000131907	0.1309	1.0028	-2.9374	0.0184	DOWN
NONMMUT064704.2	0.1307	1.0058	-2.9444	0.0310	DOWN
ENSMUST00000194646	0.0814	0.6324	-2.9578	0.0490	DOWN
NONMMUT049720.2	0.0782	0.6123	-2.9695	0.0263	DOWN
NONMMUT078645.1	0.2382	1.8772	-2.9781	0.0237	DOWN
NONMMUT117656.1	0.0561	0.4442	-2.9846	0.0459	DOWN
NONMMUT043927.2	0.0819	0.6501	-2.9883	0.0290	DOWN
NONMMUT000543.2	0.0576	0.4679	-3.0228	0.0419	DOWN
NONMMUT068748.2	0.1342	1.1011	-3.0360	0.0092	DOWN
NONMMUT029999.2	0.0716	0.5886	-3.0391	0.0367	DOWN
NONMMUT035904.2	0.1040	0.8597	-3.0477	0.0366	DOWN
NONMMUT066078.2	0.1855	1.5489	-3.0616	0.0069	DOWN
NONMMUT040063.2	0.0875	0.7428	-3.0855	0.0065	DOWN
NONMMUT013458.2	0.2930	2.4913	-3.0879	0.0045	DOWN
NONMMUT126512.1	0.1062	0.9041	-3.0892	0.0360	DOWN
NONMMUT099997.1	0.0608	0.5315	-3.1277	0.0478	DOWN
NONMMUT017212.2	0.0593	0.5213	-3.1351	0.0115	DOWN
NONMMUT100237.1	0.0638	0.5617	-3.1382	0.0338	DOWN
NONMMUT021776.2	0.0572	0.5128	-3.1633	0.0377	DOWN
NONMMUT057113.2	0.0545	0.4894	-3.1661	0.0339	DOWN
NONMMUT029827.2	0.0224	0.2055	-3.1978	0.0235	DOWN
NONMMUT029744.2	0.0738	0.6836	-3.2115	0.0204	DOWN
NONMMUT057149.2	0.0368	0.3410	-3.2117	0.0468	DOWN
NONMMUT024772.2	0.0409	0.3836	-3.2305	0.0427	DOWN
NONMMUT093987.1	0.0287	0.2708	-3.2398	0.0188	DOWN
NONMMUT017616.2	0.0990	0.9690	-3.2909	0.0402	DOWN
ENSMUST00000194957	0.1947	1.9103	-3.2949	0.0224	DOWN
ENSMUST00000200872	0.0542	0.5326	-3.2968	0.0108	DOWN
NONMMUT078786.1	0.0857	0.8452	-3.3012	0.0153	DOWN
NONMMUT069684.2	0.0322	0.3175	-3.3021	0.0395	DOWN

NONMMUT128604.1	0.1523	1.5025	-3.3025	0.0468	DOWN
NONMMUT060364.2	0.0603	0.5987	-3.3108	0.0178	DOWN
NONMMUT041854.2	0.0684	0.6902	-3.3346	0.0286	DOWN
NONMMUT054379.2	0.2755	2.7957	-3.3432	0.0038	DOWN
NONMMUT009462.2	0.0348	0.3547	-3.3477	0.0400	DOWN
NONMMUT041855.2	0.1078	1.0974	-3.3478	0.0485	DOWN
NONMMUT003929.2	0.1738	1.7815	-3.3577	0.0332	DOWN
ENSMUST00000067917	1.2336	12.7417	-3.3686	0.0125	DOWN
NONMMUT082269.1	0.1319	1.3628	-3.3690	0.0467	DOWN
NONMMUT069931.2	0.0868	0.9052	-3.3820	0.0185	DOWN
NONMMUT033200.2	0.0830	0.8752	-3.3987	0.0127	DOWN
NONMMUT048788.2	0.0351	0.3712	-3.4040	0.0113	DOWN
NONMMUT025543.2	0.1782	1.8912	-3.4078	0.0284	DOWN
NONMMUT113680.1	0.0794	0.8537	-3.4265	0.0288	DOWN
NONMMUT000579.2	0.4059	4.4469	-3.4536	0.0096	DOWN
NONMMUT001246.2	0.0265	0.2924	-3.4650	0.0329	DOWN
NONMMUT029850.2	0.0680	0.7518	-3.4674	0.0410	DOWN
MSTRG.139714.1	0.3888	4.3100	-3.4707	0.0071	DOWN
NONMMUT136706.1	0.0575	0.6401	-3.4773	0.0363	DOWN
NONMMUT116945.1	0.0193	0.2185	-3.4994	0.0312	DOWN
NONMMUT026326.2	0.0509	0.5847	-3.5233	0.0371	DOWN
NONMMUT057075.2	0.0122	0.1409	-3.5243	0.0361	DOWN
NONMMUT006099.2	0.0610	0.7068	-3.5337	0.0123	DOWN
NONMMUT024988.2	0.0155	0.1800	-3.5343	0.0448	DOWN
NONMMUT132216.1	0.3575	4.1504	-3.5371	0.0387	DOWN
NONMMUT036971.2	0.0312	0.3659	-3.5536	0.0361	DOWN
NONMMUT029174.2	0.0418	0.4916	-3.5575	0.0123	DOWN
NONMMUT103446.1	0.0601	0.7091	-3.5597	0.0032	DOWN
NONMMUT055001.2	0.0370	0.4389	-3.5702	0.0101	DOWN
NONMMUT055162.2	0.0249	0.2988	-3.5828	0.0312	DOWN
MSTRG.15948.1	0.3993	4.8456	-3.6010	0.0136	DOWN
NONMMUT051661.2	0.1630	1.9824	-3.6040	0.0363	DOWN
NONMMUT118273.1	0.1595	1.9499	-3.6115	0.0424	DOWN
ENSMUST00000192402	0.0327	0.4017	-3.6185	0.0464	DOWN
NONMMUT024045.2	0.3238	4.0551	-3.6466	0.0397	DOWN
ENSMUST00000194526	0.0516	0.6570	-3.6709	0.0252	DOWN
ENSMUST00000141259	0.0619	0.8058	-3.7028	0.0245	DOWN
NONMMUT001854.2	0.0300	0.3928	-3.7085	0.0126	DOWN
NONMMUT051900.2	0.2845	3.7994	-3.7390	0.0221	DOWN
NONMMUT024352.2	0.0244	0.3280	-3.7472	0.0181	DOWN
NONMMUT053762.2	0.0376	0.5264	-3.8080	0.0367	DOWN
NONMMUT028232.2	0.0169	0.2392	-3.8218	0.0231	DOWN
NONMMUT045372.2	0.0579	0.8247	-3.8316	0.0020	DOWN
NONMMUT098430.1	0.0651	0.9287	-3.8354	0.0423	DOWN
NONMMUT034762.2	0.0537	0.8396	-3.9661	0.0050	DOWN
MSTRG.168689.1	0.6146	9.6838	-3.9778	0.0025	DOWN
NONMMUT124386.1	0.1309	2.0701	-3.9830	0.0331	DOWN
NONMMUT005890.2	0.0329	0.5328	-4.0155	0.0244	DOWN
NONMMUT005896.2	0.0090	0.1541	-4.0952	0.0490	DOWN

NONMMUT013913.2	0.0305	0.5235	-4.0998	0.0100	DOWN
NONMMUT056492.2	0.0199	0.3426	-4.1087	0.0110	DOWN
NONMMUT001891.2	0.0921	1.6094	-4.1265	0.0123	DOWN
NONMMUT063578.2	0.0210	0.3685	-4.1304	0.0375	DOWN
NONMMUT009160.2	0.0326	0.5722	-4.1335	0.0088	DOWN
NONMMUT071926.2	0.0097	0.1721	-4.1432	0.0486	DOWN
NONMMUT037559.2	0.0157	0.2799	-4.1541	0.0433	DOWN
NONMMUT031377.2	0.1151	2.0745	-4.1712	0.0139	DOWN
NONMMUT036285.2	0.0779	1.4137	-4.1811	0.0086	DOWN
NONMMUT130248.1	0.0187	0.3505	-4.2271	0.0153	DOWN
NONMMUT065939.2	0.0097	0.1827	-4.2300	0.0331	DOWN
NONMMUT001209.2	0.0407	0.7740	-4.2490	0.0332	DOWN
NONMMUT009074.2	0.0422	0.8147	-4.2721	0.0240	DOWN
NONMMUT041169.2	0.0171	0.3318	-4.2761	0.0463	DOWN
NONMMUT050360.2	0.0461	0.9079	-4.2985	0.0034	DOWN
NONMMUT083050.1	0.0596	1.1798	-4.3072	0.0156	DOWN
NONMMUT081365.1	0.0572	1.1318	-4.3076	0.0268	DOWN
NONMMUT010137.2	0.0457	0.9312	-4.3491	0.0269	DOWN
ENSMUST00000191396	0.0475	0.9705	-4.3536	0.0366	DOWN
NONMMUT120325.1	0.0037	0.0783	-4.4148	0.0365	DOWN
NONMMUT097746.1	0.0538	1.1802	-4.4558	0.0099	DOWN
MSTRG.86142.1	0.1197	2.6563	-4.4723	0.0322	DOWN
NONMMUT081508.1	0.0360	0.8244	-4.5162	0.0437	DOWN
NONMMUT000539.2	0.0342	0.8007	-4.5510	0.0452	DOWN
NONMMUT114004.1	0.0206	0.4961	-4.5917	0.0410	DOWN
NONMMUT059853.2	0.1033	2.5422	-4.6209	0.0006	DOWN
NONMMUT047576.2	0.0186	0.4587	-4.6235	0.0437	DOWN
NONMMUT049806.2	0.0553	1.3916	-4.6524	0.0001	DOWN
NONMMUT111901.1	0.0078	0.2009	-4.6790	0.0432	DOWN
NONMMUT081301.1	0.0100	0.2581	-4.6846	0.0426	DOWN
NONMMUT040526.2	0.0133	0.3483	-4.7138	0.0454	DOWN
NONMMUT011557.2	0.0239	0.6274	-4.7155	0.0492	DOWN
NONMMUT027628.2	0.0155	0.4185	-4.7565	0.0368	DOWN
MSTRG.143441.1	0.1472	4.1287	-4.8099	0.0271	DOWN
NONMMUT061832.2	0.0195	0.5469	-4.8122	0.0184	DOWN
NONMMUT065142.2	0.0221	0.6266	-4.8267	0.0078	DOWN
NONMMUT038760.2	0.1456	4.1403	-4.8294	0.0201	DOWN
NONMMUT034030.2	0.0575	1.6649	-4.8546	0.0000	DOWN
NONMMUT111722.1	0.0318	0.9400	-4.8858	0.0294	DOWN
NONMMUT098986.1	0.0074	0.2203	-4.8909	0.0135	DOWN
NONMMUT054740.2	0.4085	12.1903	-4.8994	0.0021	DOWN
NONMMUT027068.2	0.0167	0.5098	-4.9355	0.0008	DOWN
NONMMUT030749.2	0.0068	0.2165	-4.9881	0.0489	DOWN
ENSMUST00000195730	0.0212	0.6747	-4.9951	0.0250	DOWN
ENSMUST00000202512	0.0121	0.3875	-5.0000	0.0231	DOWN
NONMMUT131241.1	0.0064	0.2045	-5.0046	0.0334	DOWN
NONMMUT129738.1	0.0171	0.5519	-5.0134	0.0406	DOWN
NONMMUT008066.2	0.0243	0.7887	-5.0222	0.0003	DOWN
NONMMUT005406.2	0.0269	0.8824	-5.0355	0.0222	DOWN

NONMMUT003759.2	0.0099	0.3257	-5.0370	0.0196	DOWN
ENSMUST00000198446	0.0063	0.2092	-5.0539	0.0131	DOWN
NONMMUT110195.1	0.0139	0.4695	-5.0794	0.0005	DOWN
NONMMUT086075.1	0.0270	0.9192	-5.0892	0.0016	DOWN
NONMMUT045184.2	0.0046	0.1611	-5.1222	0.0425	DOWN
MSTRG.123337.1	0.0638	2.2291	-5.1266	0.0294	DOWN
ENSMUST00000192642	0.0152	0.5374	-5.1432	0.0395	DOWN
NONMMUT117407.1	0.0069	0.2444	-5.1454	0.0331	DOWN
NONMMUT119200.1	0.0755	2.8037	-5.2153	0.0050	DOWN
NONMMUT011322.2	0.0309	1.1676	-5.2391	0.0012	DOWN
NONMMUT129584.1	0.0024	0.0926	-5.2483	0.0186	DOWN
NONMMUT028274.2	0.0076	0.2927	-5.2732	0.0232	DOWN
NONMMUT034724.2	0.0217	0.8642	-5.3133	0.0103	DOWN
NONMMUT107207.1	0.0328	1.3884	-5.4045	0.0211	DOWN
NONMMUT011501.2	0.0430	1.8263	-5.4078	0.0069	DOWN
NONMMUT064743.2	0.0134	0.5940	-5.4719	0.0339	DOWN
ENSMUST00000181898	0.0106	0.4794	-5.4995	0.0329	DOWN
NONMMUT062730.2	0.0054	0.2453	-5.5046	0.0258	DOWN
NONMMUT052024.2	0.0128	0.5841	-5.5080	0.0019	DOWN
NONMMUT006373.2	0.0218	1.0190	-5.5459	0.0477	DOWN
NONMMUT025322.2	0.0063	0.3115	-5.6235	0.0426	DOWN
NONMMUT005733.2	0.0061	0.3037	-5.6260	0.0335	DOWN
NONMMUT096185.1	0.0253	1.2526	-5.6277	0.0345	DOWN
ENSMUST00000205692	0.0139	0.6905	-5.6385	0.0292	DOWN
ENSMUST00000203736	0.0074	0.3721	-5.6430	0.0181	DOWN
NONMMUT024390.2	0.0038	0.1977	-5.7057	0.0077	DOWN
NONMMUT111637.1	0.0038	0.2028	-5.7458	0.0083	DOWN
NONMMUT014581.2	0.0082	0.4444	-5.7629	0.0461	DOWN
NONMMUT127924.1	0.0033	0.1856	-5.8065	0.0460	DOWN
ENSMUST00000193349	0.0053	0.3042	-5.8466	0.0489	DOWN
NONMMUT048712.2	0.0098	0.5714	-5.8678	0.0314	DOWN
NONMMUT068415.2	0.0098	0.5813	-5.8859	0.0209	DOWN
ENSMUST00000138207	0.0050	0.2991	-5.8940	0.0168	DOWN
NONMMUT130961.1	0.0049	0.3007	-5.9468	0.0105	DOWN
ENSMUST00000195353	0.0063	0.3961	-5.9805	0.0291	DOWN
ENSMUST00000192728	0.0048	0.3104	-6.0270	0.0137	DOWN
NONMMUT063759.2	0.0133	0.9124	-6.1035	0.0037	DOWN
NONMMUT011937.2	0.0082	0.5729	-6.1314	0.0338	DOWN
ENSMUST00000195293	0.0073	0.5210	-6.1517	0.0105	DOWN
NONMMUT041143.2	0.0121	0.9269	-6.2623	0.0040	DOWN
NONMMUT118818.1	0.0067	0.5626	-6.3834	0.0036	DOWN
ENSMUST00000180725	0.0077	0.6532	-6.4017	0.0176	DOWN
NONMMUT050671.2	0.0062	0.7129	-6.8563	0.0296	DOWN
ENSMUST00000172202	0.0090	1.0689	-6.8853	0.0210	DOWN
NONMMUT016434.2	0.0048	0.5890	-6.9525	0.0000	DOWN
NONMMUT100091.1	0.0038	0.4679	-6.9553	0.0004	DOWN
NONMMUT007395.2	0.0116	1.7147	-7.2074	0.0044	DOWN
